# A new gene signature for endothelial senescence identifies self‐RNA sensing by retinoic acid‐inducible gene I as a molecular facilitator of vascular aging

**DOI:** 10.1111/acel.14240

**Published:** 2024-06-21

**Authors:** Jasenka Guduric‐Fuchs, Edoardo Pedrini, Pietro M. Bertelli, Shannon McDonnell, Varun Pathak, Kiran McLoughlin, Christina L. O'Neill, Alan W. Stitt, Reinhold J. Medina

**Affiliations:** ^1^ Wellcome‐Wolfson Institute for Experimental Medicine, School of Medicine, Dentistry, and Biomedical Sciences, Queen's University Belfast Belfast UK; ^2^ Center for Omics Sciences (COSR) San Raffaele Scientific Institute Milan Italy; ^3^ Department of Eye and Vision Science Institute for Life Course and Medical Science, University of Liverpool Liverpool UK

**Keywords:** cardiovascular diseases, cellular senescence, endothelial cells, RNA sensing, senescence‐associated secretory phenotype

## Abstract

The number of senescent vascular endothelial cells increases during aging and their dysfunctional phenotype contributes to age‐related cardiovascular disease. Identification of senescent cells is challenging as molecular changes are often tissue specific and occur amongst clusters of normal cells. Here, we established, benchmarked, and validated a new gene signature called EndoSEN that pinpoints senescent endothelial cells. The EndoSEN signature was enriched for interferon‐stimulated genes (ISG) and correlated with the senescence‐associated secretory phenotype (SASP). SASP establishment is classically attributed to DNA damage and cyclic GMP–AMP synthase activation, but our results revealed a pivotal role for RNA accumulation and sensing in senescent endothelial cells. Mechanistically, we showed that endothelial cell senescence hallmarks include self‐RNA accumulation, RNA sensor RIG‐I upregulation, and an ISG signature. Moreover, a virtual model of RIG‐I knockout in endothelial cells underscored senescence as a key pathway regulated by this sensor. We tested and confirmed that RIG‐I knockdown was sufficient to extend the lifespan and decrease the SASP in endothelial cells. Taken together, our evidence suggests that targeting RNA sensing is a potential strategy to delay vascular aging.

## Graphical abstract

Establishment and validation of a new gene signature EndoSEN to identify senescent endothelial cells. An interferon‐stimulated gene signature is a hallmark of senescent endothelial cells. RNA sensor RIG‐I is activated in senescent endothelial cells. Human senescent endothelial cells accumulate endogenous self‐RNA. Knockdown of RIG‐I extended the lifespan and decreased the inflammatory phenotype (SASP) in aged endothelial cells.

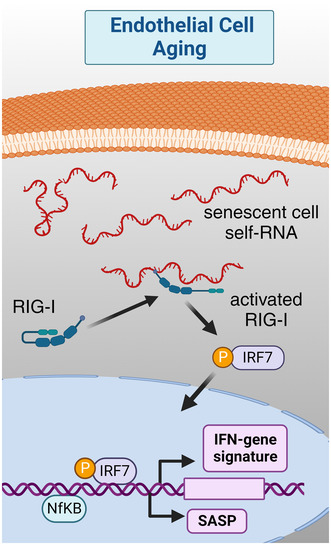

**Abbreviations**
ECFCs: endothelial colony forming cells
EP: early passage
Eto: etoposide
GSEA: gene set enrichment analysis
ISG: interferon‐stimulated gene
ISM: interferon signature metric
ISRE: interferon stimulated response element
MP: mid passage
NES: normalized enrichment score
Rep: replicative
RIG‐I: retinoic acid‐inducible gene I
SASP: senescence‐associated secretory phenotype
SA‐β‐Gal: senescence‐associated beta‐galactosidase
SCPA: single cell pathway analysis
Sen: senescent

## 1 INTRODUCTION

Cardiovascular aging is associated with an increased prevalence of atherosclerosis, hypertension, and myocardial infarction (Sun, 2015). Endothelial cell senescence and the resultant loss of homeostasis has been implicated in age‐related cardiovascular pathology (Childs et al., 2018) with associated thrombotic and pro‐inflammatory events leading to inadequate tissue perfusion (Bochenek et al., 2016). Senescent cell‐clearing senolytic therapy has been shown to improve age‐related vascular pathology in a murine model (Roos et al., 2016). These findings highlight a role for cellular aging in cardiovascular disease, however further research will require new strategies to identify senescent cells, as this is recognized as a major bottleneck for senescence research (Gil, 2023).

Cellular senescence is a permanent state of cell cycle arrest characterized by multiple phenotypic changes, including impaired functionality (Gorgoulis et al., 2019). According to tissue and disease context, senescence is induced by replicative stress, oncogene activation, or genotoxic stress. Hallmarks for the senescence phenotype include positivity for senescence‐associated beta‐galactosidase (SA‐β‐Gal), DNA damage, and the senescence‐associated secretory phenotype (SASP) (Hernandez‐Segura et al., 2018). The SASP is a complex secretome produced by senescent cells, composed of cytokines, chemokines, bioactive lipids, and damage‐associated molecular patterns (Wiley & Campisi, 2021). DNA damage and the SASP have been linked by the finding that cytoplasmic chromatin fragments in senescent cells trigger the activation of DNA‐sensing receptor cyclic GMP–AMP synthase (cGAS) pathway and induce the secretion of inflammatory cytokines (Glück et al., 2017; Lan et al., 2019). cGAS induces interferon‐stimulated genes (ISGs) and reports have identified ISGs in senescent cells (De Cecco et al., 2019; Kreienkamp et al., 2018). Therefore, the innate immune response linked to senescence, has been attributed to cytoplasmic DNA sensing (Di Micco, 2017). On the contrary, RNA sensing during senescence remains largely underexplored, despite the findings for accumulation of long promoter RNAs and 3′ gene in a model of oncogene‐induced senescence (Mullani et al., 2021). It is known that retinoic acid‐inducible gene I (RIG‐I) is a prototypical RNA sensor that recognizes viral RNA and triggers innate immune responses (Stok et al., 2020). Interestingly, RIG‐I is upregulated in replicative senescence and murine aging, where it promotes IL6 and IL8 expression (Liu et al., 2011). Counterintuitively, RIG‐I^−/−^ mice exhibit premature aging (Zhao et al., 2019). Such conflicting results underscore the need to elucidate the role for RNA sensor RIG‐I in cellular senescence.

In this study, we established the gene signature EndoSEN to enable molecular identification of senescent endothelial cells. In addition, we uncover a role for innate RNA sensing in cellular aging by demonstrating that senescent human endothelial cells upregulate RIG‐I coupled with an ISG signature. Furthermore, we show that, in endothelial cells, senescence is linked to self‐RNA accumulation and its binding to RIG‐I. Moreover, RIG‐I knock‐down was an effective strategy to delay senescence establishment, reduce SASP, and extend the lifespan of endothelial cells in vitro.

## 2 RESULTS

### 2.1 Defining an endothelial senescence (EndoSen) gene signature

**FIGURE 1:**
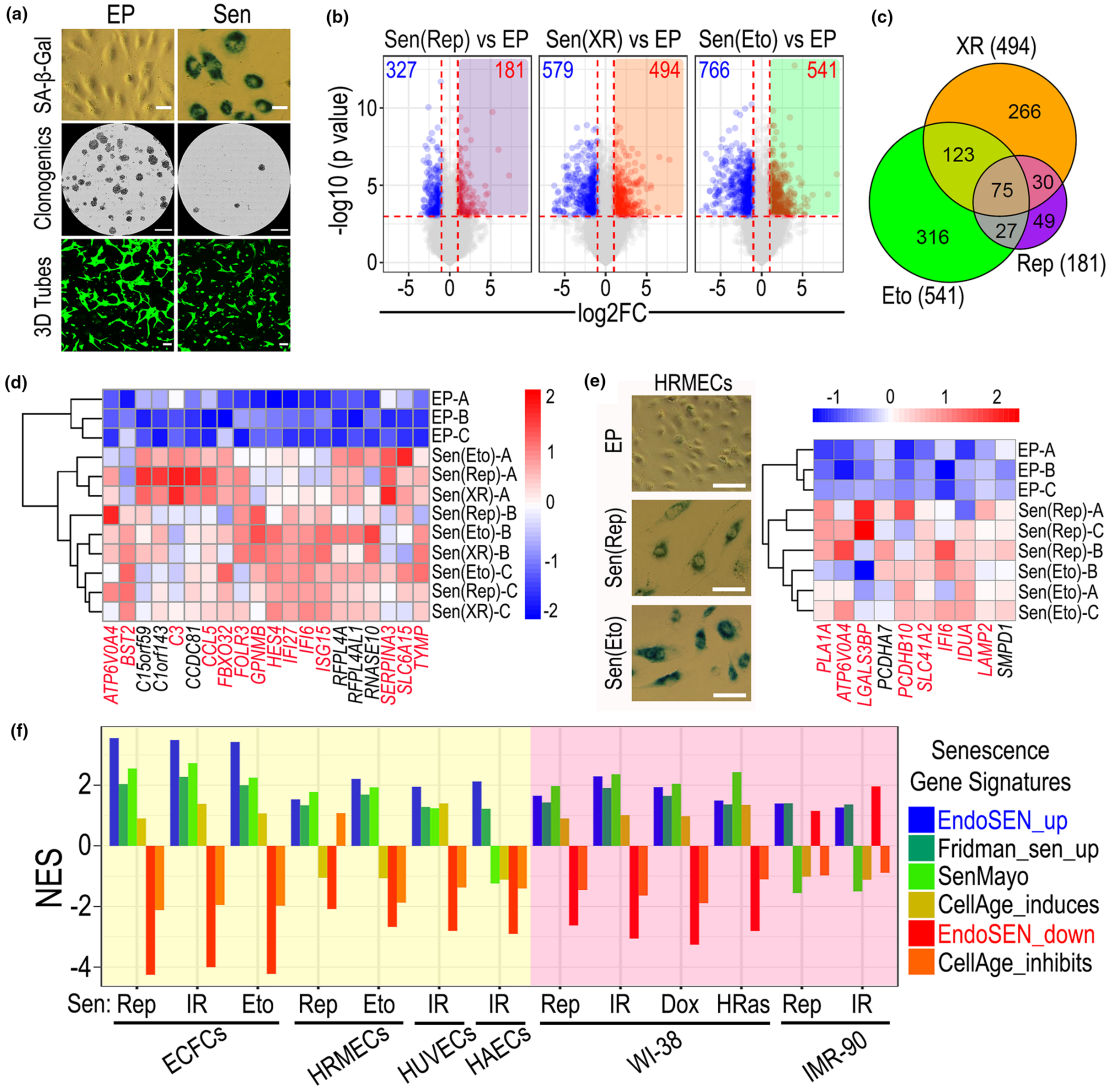
Establishing and benchmarking a senescence gene signature for endothelial cells. (a) Images of early passage (EP) and senescent (Sen) ECFCs stained for senescence‐ associated β‐Galactosidase activity (SA‐β‐Gal). Scale bar: 50 μm. Representative cell culture wells stained with crystal violet to assess for clonogenic capacity. Scale bar: 5 mm. 3D Matrigel angiogenesis assay. ECFCs were stained with calcein for fluorescent microscope imaging at Day 2. Scale bar: 100 μm. (b) Genome‐wide transcriptomics differential gene expression analysis shown in volcano plots. Significantly upregulated transcripts highlighted in purple, orange, and green. (c) Euler diagram for upregulated genes from the three cellular senescence models to identify a common senescence signature in endothelial cells. (d) Heatmap and unbiased clustering analysis for the top 20 upregulated genes based on log 2‐fold change. IFN‐related transcripts are highlighted in red. (e) The images of EP, replicative (Rep) Sen, and etoposide (Eto)‐induced Sen; human retinal microvascular endothelial cells (HRMECs) stained for SA‐β‐Gal. Scale bar: 100 μm. Heatmap and unbiased clustering analysis, using the EndoSEN_up gene signature, showing the top 10 upregulated genes in Sen HRMECs compared to EP HRMECs. (f) Normalized enrichment score (NES) for GSEA benchmarking our EndoSEN signatures to previously established senescence gene signatures. Signatures were assessed across datasets for endothelial cells (yellow background) and fibroblasts (pink background). ECFCs, endothelial colony forming cells; HAECs, human aortic endothelial cells; HRMECs, human retinal microvascular endothelial cells; HUVECs, human umbilical vein endothelial cells; WI‐38 and IMR‐90, human lung fibroblasts.

To investigate cell senescence, we employed human cord blood‐derived primary endothelial colony forming cells (ECFCs), because of their high replicative potential, remarkable purity, and gradual in vitro establishment of senescence (Medina et al., 2013). These attributes enabled consistent modeling of endothelial cell senescence. We induced cellular senescence in human cord blood‐derived ECFCs from three donors using three well‐accepted approaches: serial passage until Hayflick limit, also known as replicative senescence (Rep), DNA damage stress‐induced senescence using X‐rays or treatment with Etoposide (Eto). Senescence was confirmed by staining for SA‐β‐Gal biomarker, clonogenic assay to demonstrate cell cycle arrest, and in vitro 3D angiogenesis to show endothelial cell functional impairment (Figure 1a and Figure S1). Unbiased genome‐wide transcriptomic analysis was performed, and differential gene expression identified 181, 494, and 541 transcripts significantly upregulated in the Rep, X‐rays, and Eto‐induced senescent cells respectively (Figure 1b). To determine a common endothelial cell senescence gene signature irrespective of the inducer, we integrated the results from the three senescence models and identified 75 genes commonly upregulated (Figure 1c). These 75 genes upregulated in the three approaches of induced endothelial senescence were named EndoSEN_up gene signature. Similarly, we identified 209 genes that were commonly downregulated and named EndoSEN_down gene signature (Figure S2a). Unsupervised hierarchical clustering using the EndoSEN_up gene signature showed the effectiveness of this gene signature for separating senescent from early passage (EP) cells, and the heatmap visualization of the top 20 genes, based on average log2 fold change showed compelling differences between EP and senescent cells (Figure 1d). In addition, interestingly, 14 out of the top 20 genes are included in the Interferome database, and are highlighted in red. The Interferome database is a comprehensive online resource of validated interferon‐regulated genes (Rusinova et al., 2013). Importantly, the Interferome database is open access; therefore, we harnessed its information to identify interferon associated genes in our data. As expected, there are some differences depending on the cell senescence model used, but when visualizing the top 20 differentially expressed genes in ECFCs, we observed a remarkable similarity in p values and fold change across the models (Figure S3). To evaluate the applicability of EndoSEN_up gene signature to other human endothelial cells, we repeated the senescence‐induction experiment using human retinal microvascular endothelial cells (HRMECs). HRMECs were chosen because it was recently reported that senescent HRMECs exhibit SASP associated with nucleic acid sensing (Liu et al., 2023). Senescence in HRMECs was confirmed by SA‐β‐Gal staining (Figure 1e), in addition to cell cycle arrest and decreased tubulogenic potential (Figure S4). RNA‐seq confirmed that the EndoSEN_up gene signature effectively separated EP from senescent HRMECs (Figure 1e). From the top 10 upregulated genes, 8 are found in the Interferome database and highlighted in red. While GSEA plots showed a positive enrichment for our EndoSEN_up signature in ECFCs and HRMECs, the normalized enrichment scores (NES) were higher for ECFCs (Figure S5a). There were some differences in the distribution of the genes across the leading edges of the signature, depending on the model and cell type (Figure S5b). Furthermore, we benchmarked EndoSEN_up or EndoSEN_down against currently used cell senescence gene signatures Fridman, SenMayo, and CellAge, using additional publicly available data for human umbilical vein endothelial cells (HUVECs), human aortic endothelial cells (HAECs), and human fibroblast cell lines WI‐38 and IMR‐90. Details for the gene signatures used are available in supplementary files (Table S1). Results from the comparison of EndoSEN_up with other senescence signatures, highlighted that the NES was higher for EndoSEN_up than for Fridman_up, SenMayo, and CellAge_induces, when assessing endothelial cells (Figure 1f). Similarly, negative NES for EndoSEN_down was lower than for CellAge_inhibits. These higher scores from our new EndoSEN signatures, when compared to others, were not seen when assessing senescent fibroblasts. Taken together, we have established and validated an endothelial cell senescence gene signature EndoSEN, which showed higher NES scores compared against current standard senescence gene signatures, when evaluating the senescence status of endothelial cells.

### 2.2 Interferon‐related genes are a hallmark of endothelial senescence

**FIGURE 2:**
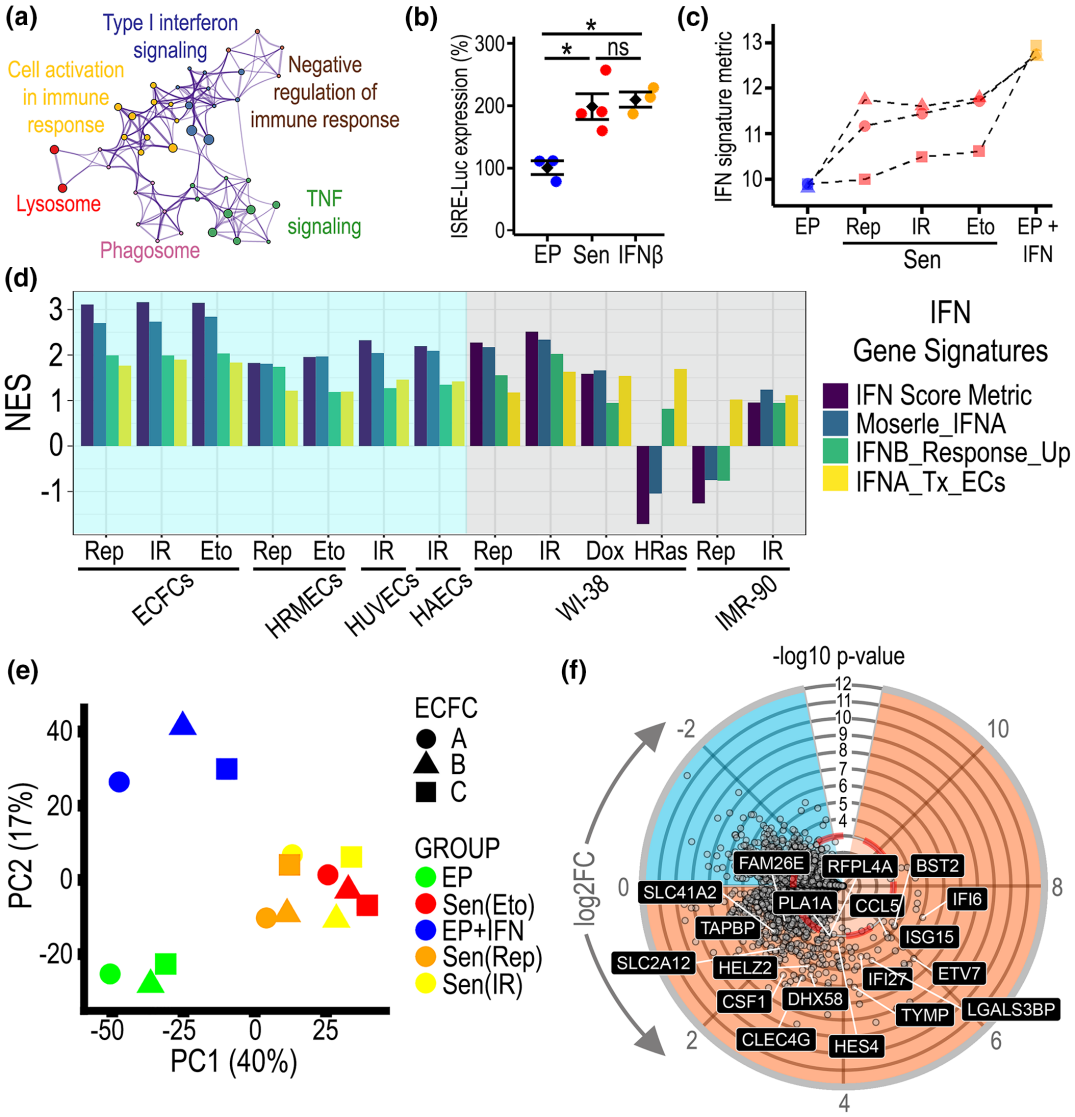
Senescent endothelial cells exhibit an interferon‐stimulated gene (ISG) signature. (a) Metascape pathway network analysis from EndoSEN_up signature. (b) Interferon Stimulated Response Element (ISRE) promoter luciferase reporter system used to assess IFN signaling bioactivity in EP and Sen ECFCs from the three models. ECFCs treated with 100 IU/mL IFNβ were used as positive controls and shown in orange. *N* = 4, ns: not significant, *p < 0.05. (c) Comparison of IFN signature metric (ISM) across experimental groups in three biological replicates. (d) Normalized enrichment score (NES) for interferon gene signatures from MSigDB in our and other publicly available datasets. Signatures were assessed across endothelial cells (light blue background) and fibroblasts (grey background). (e) Principal component analysis of transcriptomes obtained from five experimental groups (EP, IFN‐treated EP, and the three senescence models Replicative, Etoposide, and X‐rays), each from three biological replicates. (f) Circular volcano plot for IFN‐treated ECFCs depicting upregulated genes in orange and downregulated in light blue. The genes that are common to EndoSEN_up have been highlighted and named.

To identify molecular pathways from the EndoSEN_up gene signature, we performed Metascape Pathway analysis, which identified Interferon as the second most enriched term in EndoSEN_up after lysosome. The Metascape pathway network showed Type I interferon signaling at the center of the semantic space and interacting with immune response and TNF (Figure 2a). In addition, we performed EnrichR‐Reactome pathway analysis. Among the ten most enriched pathways in EndoSEN_up, three of them were related to Interferon (Table 1). This was also visualized in the Reactome‐based pathway network which highlighted interferon signaling among the largest subnetworks (Figure S6). This suggested ISGs as a feature of endothelial senescence. To monitor interferon signaling activation, we used the Interferon Stimulated Response Element (ISRE) reporter system and found that the luminescence reporter values were significantly increased in senescence when compared to EP cells, and not‐different from the positive control interferon‐treated cells (Figure 2b). To further confirm ISGs in endothelial senescence, we used a validated Interferon signature metric (ISM) established for categorizing patients with systemic lupus erythematosus (Kennedy et al., 2015). We found that the ISM increased in the three senescence models with values closer to the positive control (Figure 2c). In addition, using publicly available senescence transcriptome datasets for human endothelial cells and fibroblasts, and applying validated interferon gene signatures from the Molecular Signatures Database (Table S1), we found that senescent endothelial cells but not fibroblasts, had consistent positive NES (Figure 2d). To uncouple the acute IFN response from the senescence‐associated IFN signature and to identify potential molecular drivers, we performed transcriptomics including an Interferon‐treated experimental group. The dimensionality reduction analysis using PCA separated senescent from EP cells in PC1, as expected; while PC2 segregated experimental groups based on the interferon treatment (Figure 2e). Interferon treatment induced more gene upregulation (orange) than downregulation (blue) as seen in the circular volcano plot. Within the interferon‐induced endothelial gene signature, we identified 19 genes that were also differentially expressed in endothelial senescence (Figure 2f). This analysis also identified 8 genes that were commonly downregulated in both the senescence and IFN‐treated experimental groups (Figure S2b). To pinpoint a master regulator for these ISGs in endothelial senescence, we evaluated the transcription factor co‐expression for the common 19 upregulated genes using EnrichR ARCH4 TF. This revealed *IRF7* as the transcription factor associated with the highest number of genes from the signature (Figure S7). These results verified ISGs as a molecular fingerprint of endothelial senescence.

**TABLE 1:** Output table from pathway analysis for commonly upregulated genes in senescent ECFCs (EndoSEN_up signature) using the REACTOME 2022 database and EnrichR software. Three gene signatures in the top 12 are associated to interferon signaling and 4 gene signatures in the top 12 are associated with the immune system and cytokines.

Rank	ID	Term	*p*‐value	Adjusted *p*‐value	Odds ratio	Combined score
1	R‐HSA‐913531	Interferon signaling	1.00542E‐05	0.0012	10.52	121.11
2	R‐HSA‐168256	Immune system	5.88957E‐06	0.0012	3.64	43.87
3	R‐HSA‐909733	Interferon alpha/beta signaling	0.000154698	0.0127	16.45	144.35
4	R‐HSA‐6783783	Interleukin‐10 signaling	0.000641775	0.0396	19.73	145.01
5	R‐HSA‐1280215	Cytokine signaling in immune system	0.001219449	0.0602	3.78	25.39
6	R‐HSA‐74752	Signaling by insulin receptor	0.003036354	0.1250	11.18	64.80
7	R‐HSA‐77387	Insulin receptor recycling	0.004254161	0.1409	22.72	124.04
8	R‐HSA‐877300	Interferon gamma signaling	0.004565111	0.1409	9.61	51.80
9	R‐HSA‐168249	Innate immune system	0.005141528	0.1411	2.84	14.95
10	R‐HSA‐917977	Transferrin endocytosis and recycling	0.006013544	0.1450	18.80	96.12
11	R‐HSA‐5663084	Diseases of carbohydrate metabolism	0.006795357	0.1450	17.58	87.76
12	R‐HSA‐1222556	ROS and RNS production in phagocytes	0.008049561	0.1450	16.03	77.29

### 2.3 ISGs are upregulated in mouse retinal endothelial cells with age

**FIGURE 3:**
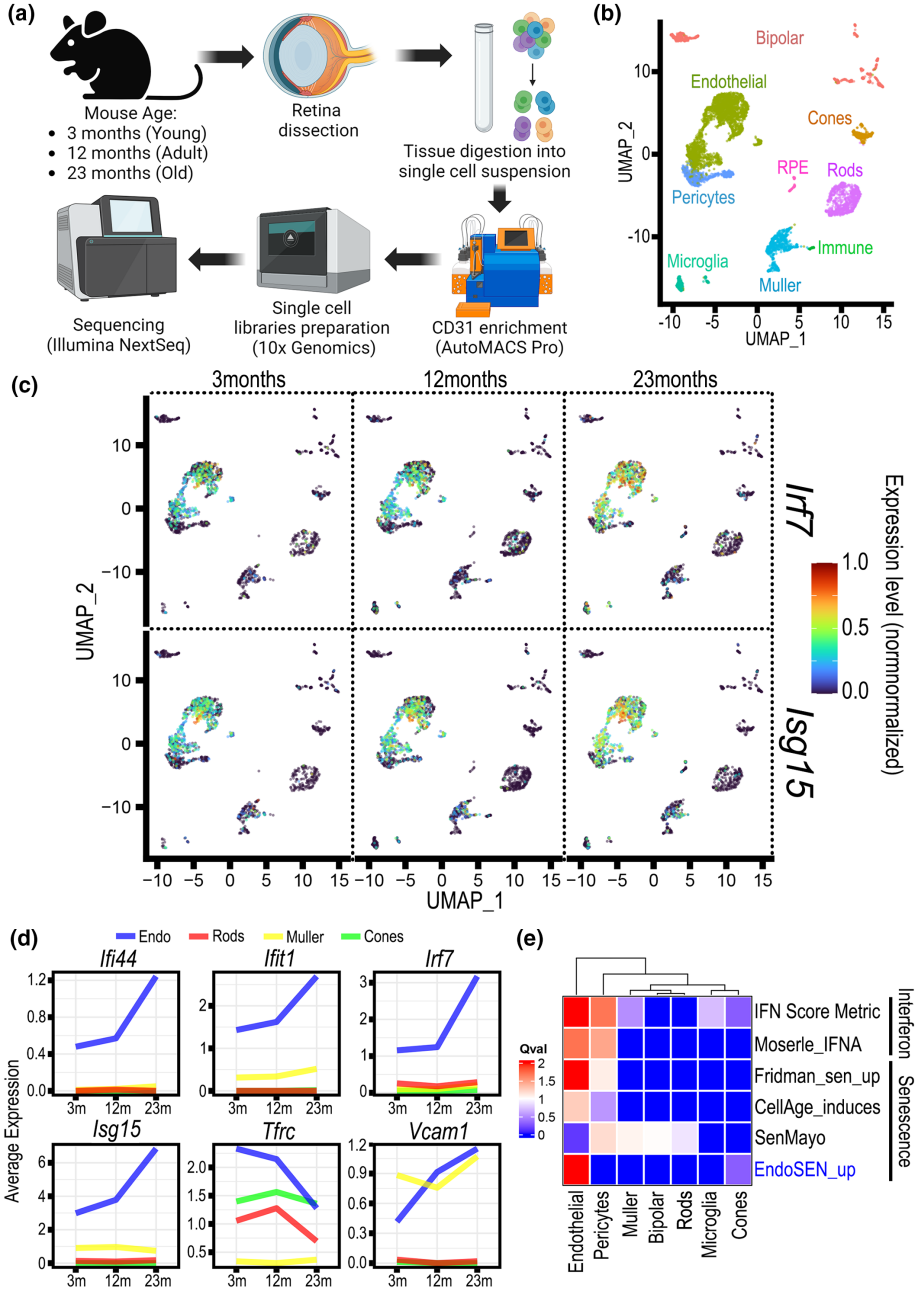
Endothelial cells in the aged mouse retina show an ISG and EndoSEN gene signatures. (a) Experimental design. Cell suspensions for scRNA‐seq were obtained from mouse retinas at 3 ages: 3, 12, and 23 months. Enrichment for CD31+ cells was performed using the autoMACS. Created with BioRender.com. (b) UMAP visualization of distinct retinal cell types isolated from mouse retinas. (c) Gene expression level depicted in UMAPs for Irf7 and Isg15 across the three age groups. (d) Average expression level for interferon related genes across endothelial cells, rods, Muller cells, and cones. (e) Heatmap depicting results from Single Cell Pathway Analysis to evaluate interferon and senescence gene signatures in each cell cluster.

To evaluate the interferon‐related gene signature in the aging vasculature at single cell resolution in vivo, we performed scRNA‐seq in retinas from 3‐, 12‐, and 23‐month‐old mice as equivalents for young, middle‐aged, and old in human age (Flurkey et al., 2007). We isolated single cells from retinal tissue and employed the autoMACS system with a CD31 antibody to enrich for endothelial cells from <1% in original sample to ≥40% in the positive sorted fraction (Figure 3a). Samples were integrated and batch‐effect corrected using Seurat V3 algorithms. We excluded barcodes that expressed less than 500 genes or more than 6000. We also excluded barcodes with more than 20% mitochondrial reads and less than 1% mitochondrial reads, because they represented poor‐quality cells. Doublets were detected using DoubletFinder (McGinnis et al., 2019) and filtered out. 5332 cells passed QC criteria for further analysis, the average number of genes per cell was 2487. Cells were clustered and visualized using the Uniform Manifold Approximation and Projection (UMAP). After expert‐based annotation, we identified nine cell types (Figure 3b) based on expression of specific lineage marker genes (Figure S8). The frequency and distribution of cell types was similar across the three age groups (Figure S9); however, an increase in the expression of ISGs such as *Irf7* and *Isg15* were observed associated with aging (Figure 3c). Importantly, the level of ISG expression was the highest in the 23‐month group and was restricted to endothelial cells and not seen in other cell types such as rod and cone photoreceptors, or Muller (glial) cells (Figure 3d). In addition, we used single cell pathway analysis (SCPA) to compare gene signature enrichments scores for Interferon and senescence‐related pathways. SCPA results highlighted that the interferon signatures ISM and Moserle were upregulated in retinal endothelial cells and pericytes with age (Figure 3e). Furthermore, we used SCPA to benchmark our EndoSEN_up signature against frequently used senescence signatures Fridman, CellAge, and SenMayo. This analysis underscored the specificity of EndoSEN_up signature towards endothelial cells (Figure 3e) and confirmed their applicability in mouse retina. Here, we corroborated that the mouse retinal vasculature exhibited an interferon gene signature associated with aging and that retinal endothelial cells in aged mice were enriched for our EndoSEN_up gene signature.

### 2.4 Senescent human endothelial cells increased expression of RNA sensors coupled with self‐RNA accumulation

**FIGURE 4:**
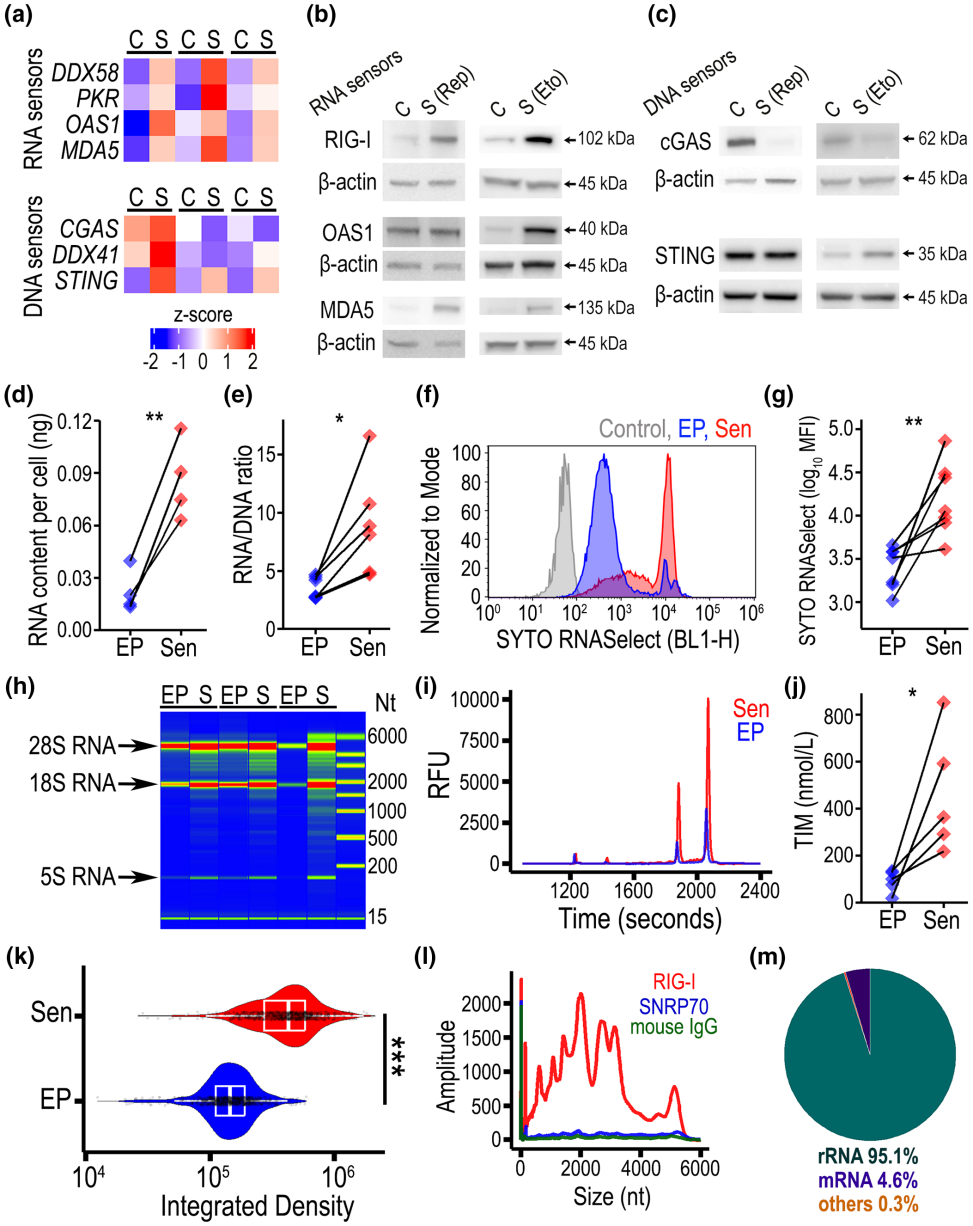
Expression of nucleic acid sensors and accumulation of self‐RNA in senescent endothelial cells. (a) Heatmap depicting z‐scores for qRT‐PCR results evaluating gene expression of RNA and DNA sensors when comparing control (C) and senescent (S) ECFCs in the replicative senescence model across three biological replicates. (b) Protein expression changes for RNA sensors RIG‐I, OAS1, and MDA5 with endothelial senescence. (c) Protein expression changes for DNA sensors cGAS and STING with endothelial senescence. (d) RNA content per cell, evaluated from total RNA extracted from 200,000 early passage (EP) or senescent (Sen) ECFCs using the Maxwell automated system in the replicative senescence model. (e) RNA/DNA ratio quantified in EP and Sen ECFCs using the replicative senescence model. (f) Representative flow cytometry histograms to depict RNA binding dye SYTO RNASelect fluorescence signal using the etoposide‐induced senescence model. (g) Quantification and statistical analysis of SYTO RNASelect results. MFI, median fluorescence intensity. (h) Density gel image from a Fragment Analyzer system assessment of total RNA separation highlighting ribosomal RNA bands, using the replicative senescence model. (i) Electropherogram overlay of a representative paired sample for EP versus replicative Sen ECFCs. (j) Dot plot comparison for the quantification of total integrated molarity (TIM) in EP and Sen ECFCs, from the Fragment Analyzer assessment. (k) Violin plots quantification of RNA synthesis evaluated using Click‐iT chemistry in the etoposide‐induced senescence model. (l) Electropherogram overlay for RIP RNA samples precipitated using RIG‐I or SNRNP70 antibodies in replicative senescence model. Mouse IgG was used as a negative control. (m) Pie chart showing the frequency of RNA species found to bind RIG‐I protein. *p < 0.05, **p < 0.01, ***p < 0.001, ns: non‐significant, *n* ≥ 4 independent experiments.

Based on our finding that senescent endothelial cells exhibited ISGs and previous reports linking nucleic acid sensing with cellular aging (Yang et al., 2017), we investigated expression of RNA and DNA sensors in EP control (C) and senescent (S) human endothelial cells. At the mRNA level, we found consistent upregulation of RNA sensors *DDX58* (RIG‐I), *PKR*, *OAS1*, and *MDA5* by RT‐qPCR (Figure 4a). This gene upregulation was not observed for DNA sensors *cGAS* and *DDX41* but remained for *STING*. The cGAS/STING gene signature M47538 from KEGG pathways was evaluated in endothelial cells from the mouse scRNAseq data. While *cGAS* gene expression was negligible, *Irf3* and *Tbk1* were expressed but no difference was found across the three ages (Figure S10). At the protein level, although there were some differences between the senescence models, increased expression of RNA sensor RIG‐I was consistent in both replicative and Etoposide‐induced senescence (Figure 4b). On the other hand, DNA sensor cGAS was consistently downregulated (Figure 4c). Quantitative analysis confirmed that in replicative senescence, there was a significant increase in RIG‐I and MDA5 (Figure S11a), while Etoposide‐induced senescence showed a significant increase in RIG‐I and OAS1 (Figure S11b). To evaluate the possibility that RIG‐I upregulation is associated with the quiescent state, we assessed RIG‐I protein expression in quiescent ECFCs in comparison to EP proliferating and etoposide‐induced senescent cells. We found that quiescent ECFCs exhibited significantly lower RIG‐I expression than senescent ECFCs (Figure S12a,b). Interestingly, in both endothelial senescence models, we found a significant downregulation of DNA sensor cGAS. Increased STING protein expression was only significant in the Etoposide‐induced senescence model. These results demonstrated that human endothelial senescence is associated with a consistent increase in RNA sensor RIG‐I expression at the transcript and protein levels. This led us to hypothesize that RIG‐I may bind endogenous self‐RNA that accumulates with cellular aging. To test this, we measured absolute and relative RNA content in EP and replicative senescent (Sen) endothelial cells. We found a significant increase in RNA content per cell in senescent cells when compared to EP cells (Figure 4d), which agreed with the significant increase in the RNA/DNA ratio (Figure 4e). Etoposide‐induced senescent cells also exhibited a significant increase in SYTO RNASelect signal by flow cytometry, which specifically labels intracellular RNA (Figure 4f,g). Furthermore, total RNA was extracted from 200,000 cells from either EP or replicative Sen experimental groups and loaded into a Fragment Analyzer System to examine RNA profiles. This further demonstrated higher RNA quantity in Sen than in EP cells, which was particularly evident at the ribosomal RNA (rRNA) bands (Figure 4h). Electropherograms showed higher peaks for Sen cells when compared to EP counterparts (Figure 4i and Figure SF13a). Quantification confirmed a significant increase in RNA total integrated molarity by 3.9‐fold, from 110 nM/L in EP to 432 nM/L in Sen cells (Figure 4j). To evaluate changes in global RNA synthesis at the single‐cell level, we used Click‐iT chemistry to detect 5‐ethynyluridine incorporation into nascent RNA transcripts. We found that RNA biosynthesis was significantly increased in etoposide‐induced Sen when compared to EP endothelial cells (Figure 4k). To directly link the self‐RNA accumulation with increased RIG‐I, we performed RIP‐Seq for RIG‐I. As expected, we confirmed the endogenous RNA binding to RNA sensor RIG‐I which was ~10‐fold higher than IgG and SNRNP70 controls (Figure 4l and Figure SF13b). Ribosomal RNAs represented the most frequent RNA species bound to RIG‐I (Figure 4m). These results were further corroborated by RIP‐qPCR that also showed a significant increase in bound rRNAs 5.8S and 18S when compared to U1 RNA, which is known to specifically binds to SNRNP70, and was used as a positive control (Figure SF13c). Specificity of the assay was also confirmed by increased binding of previously confirmed RIG‐I target *TRIM25* mRNA (Wu et al., 2020). When comparing normalized number of reads for transcripts binding RIG‐I antibody or control IgG from the RIP‐Seq data, we found a significant increase in mRNAs and rRNAs bound to RIG‐I (Figure SF14). Considering that rRNA production takes place in the nucleolus, we investigated nucleolus size by immunostaining for Nucleolin. We observed nucleolar expansion in senescent cells as shown by the increased Nucleolin stained areas in senescent when compared to EP cells (Figure SF15). All this evidence indicates that self‐RNA accumulation by increased RNA biosynthesis is a molecular feature of senescent endothelial cells that can trigger RIG‐I sensing and consequently, the IFN gene signature.

### 2.5 Computational modeling of the RIG‐I virtual knockout in human endothelial cells predicts an effect on cellular senescence

**FIGURE 5:**
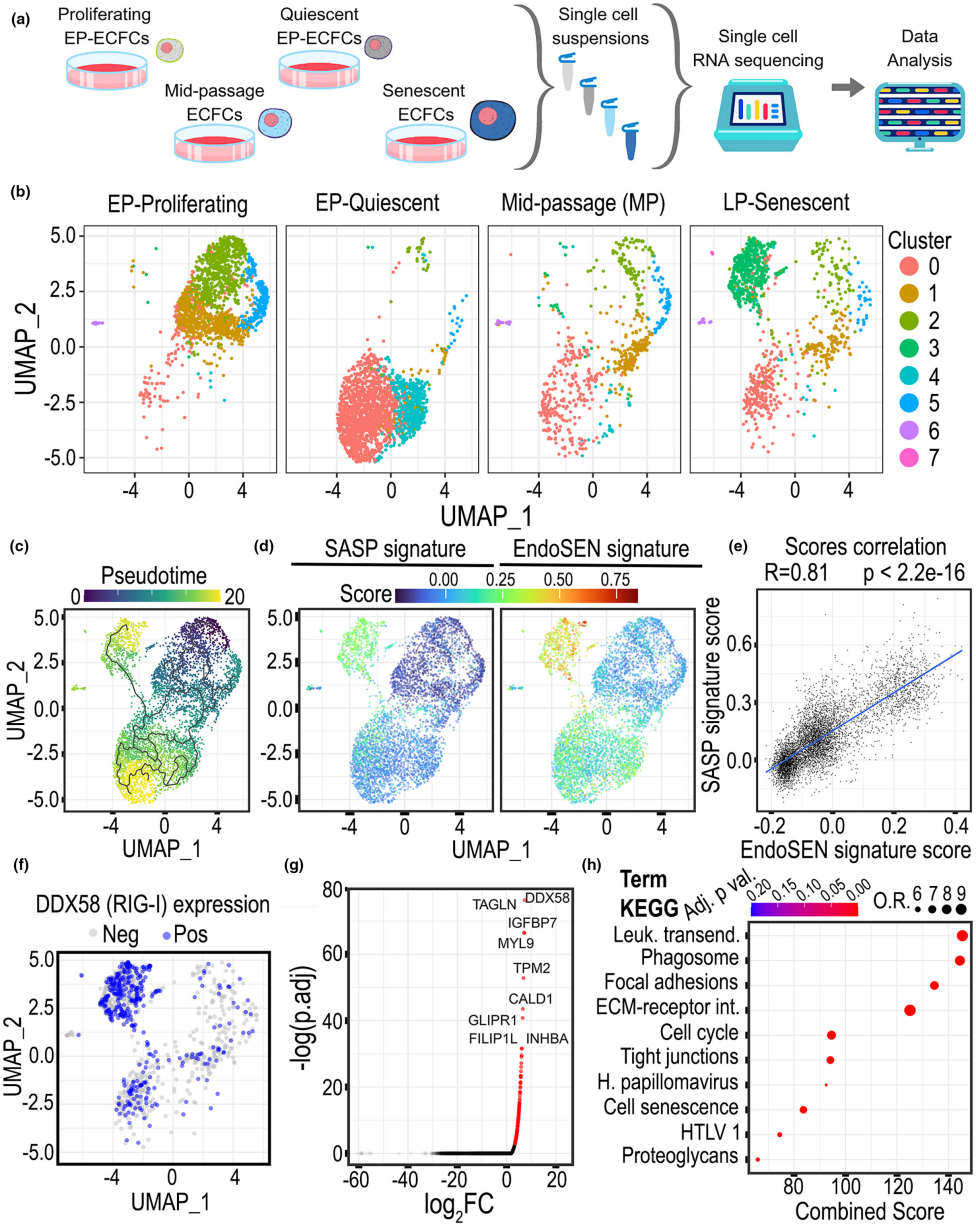
scRNA‐seq data and scTenifoldKnK computational tool predicted that the RIG‐I knockout in endothelial cells affects expression of senescence genes. (a) Experimental design. Human endothelial cells at early passage (EP) proliferating, EP quiescent, mid‐passage, and senescent were analyzed by scRNA‐seq. Created with Canva.com. (b) UMAPs to visualize the cell cluster changes across the in vitro lifespan on human endothelial cells. (c) Pseudotime analysis showing gene trajectories. (d) GSEA scores for SASP and Endosen_up gene signatures. (e) Correlation analysis for SASP and EndoSen_up gene signatures. (f) Expression level of RIG‐I positive cells in the scRNAseq dataset. (g) scTenifold virtual RIG‐I knockout. Genes that are significantly affected by the RIG‐I virtual knockout are highlighted in red. (h) KEGG pathway enrichment analysis for genes predicted to be dysregulated by the RIG‐I virtual knockout.

To gain a better understanding of the heterogeneity in the cell population during onset of the senescence process, we performed scRNA‐seq in human endothelial cells at EP, middle passage (MP), and senescent (Sen) stages (Figure 5a). We also included an EP quiescent experimental group because cell cycle arrest is an essential characteristic of senescence, but insufficient as a single indicator for this phenotype. Samples from the four experimental groups were integrated and batch‐effect corrected using Seurat V3 algorithms. We excluded barcodes that expressed less than 2000 genes or more than 9000, we also excluded barcodes with more than 10% and less than 1% mitochondrial reads. Doublets were detected using DoubletFinder and filtered out. 5696 single cells passed QC criteria for further analysis. Cells were clustered and visualized using UMAPs. We identified 8 clusters (Figure 5b). Clusters 1, 2, and 5 identified proliferating cells, while clusters 0 and 4 were the non‐cycling cells. A mixture of these clusters was present in the MP cells, and we identified cluster 3 as the senescent cluster. Pseudotime analysis was performed using Monocle (Trapnell et al., 2014) and results highlighted that the trajectory starting at proliferating endothelial cells moved through quiescence before reaching the senescence cluster (Figure 5c). In addition, we evaluated a SASP and the EndoSEN_up gene signatures, and we found both to be enriched in the senescence cluster 3 (Figure 5d). We then scored each single cell for both SASP and EndoSEN_up and performed correlation analysis. The results indicate a significant strong Pearson correlation coefficient of 0.8 between the SASP and EndoSEN_up gene signatures (Figure 5e). To further validate our EndoSEN_up signature, we applied the senescence index tool (SIT) algorithm for a binary annotation of senescent and non‐senescent cells (Troiani et al., 2022). SIT agreed with EndoSEN_up and SASP signatures in annotating cluster 3 as senescent, while other clusters were non‐senescent (Figure SF16). This senescence index tool also validated our replicative senescence model, showing that the percentage of senescent cells increased from 1% in EP‐proliferating cells, 29% in MP cells, to 77% in LP‐senescent cells. The expression of prototypical senescence genes including *CDKN2A* (p16), *CDKN2B* (p15), *CDKN1A* (p21), and *SERPINE1* (PAI1), were all significantly upregulated with senescence (Figure SF17a). At the protein level, we also observed an increase in p21 expression in both the replicative and etoposide‐induced senescence models (Figure SF18a‐b). We found that the expression of *DDX58* (RIG‐I) was the highest in the senescence cluster (Figure 5f). Detailed assessment highlighted that *DDX58* expression in quiescent ECFCs was significantly lower than in senescent ECFCs, and comparable to EP proliferating and mid passage (Figure SF12c). The cGAS/STING pathway was also evaluated, and the expression of *CGAS*, *IRF3*, and *TBK1* were significantly decreased in late passage senescent ECFCs (Figure SF17b). Then, we applied the machine learning tool ScTenifoldKnK to our endothelial scRNA‐seq data to enable the virtual knockout of *DDX58* in senescent cells (LP) and prediction of differentially regulated genes. Virtual knockout of *DDX58* was predicted to significantly alter the expression of 206 genes (Table S2), including *TAGLN*, *IGFBP7*, and *MYL9* (Figure 5g). We performed KEGG pathway analysis on this gene list and found that the *DDX58* virtual knockout model was predicted to significantly impact cellular senescence (Figure 5h). These results from scRNA‐seq of human endothelial cells corroborated the EndoSEN_up gene signature enrichment in the senescent cluster, with a high expression of *DDX58* (RIG‐I), and the virtual *DDX58* knockout model was found to have a role in the modulation of cellular senescence.

### 2.6 The RIG‐I knockdown extends the in vitro lifespan of endothelial cells and delays senescence

**FIGURE 6:**
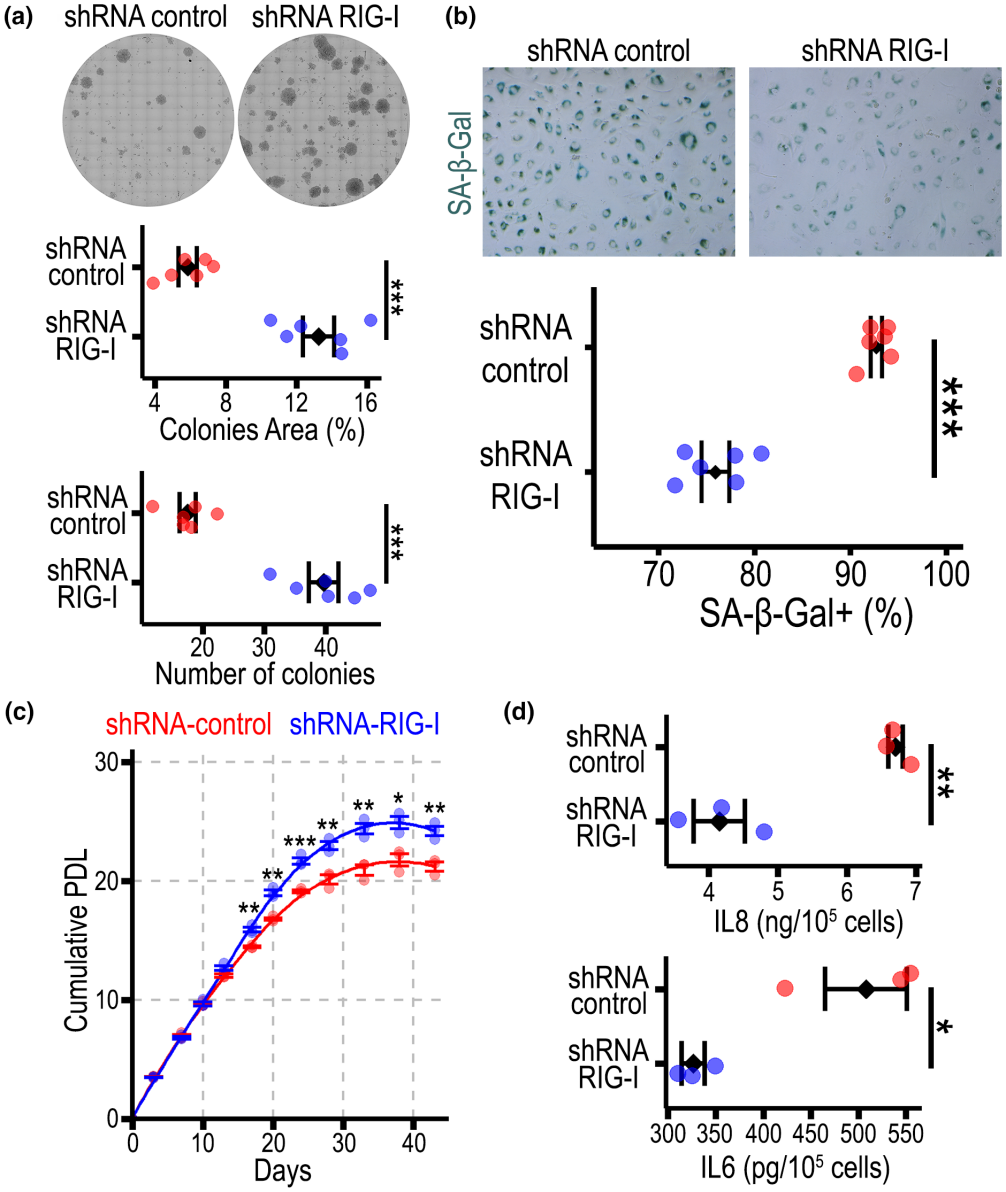
Knockdown of RIG‐I delays senescence establishment in endothelial cells. Stable RIG‐I knock down cells were produced at an early passage using RIG‐I shRNA lentiviral vector. Control cells were transduced with a same vector containing nontargeting control shRNA. After confirming RIG‐I knockdown, senescence was induced by serial passaging or Etoposide exposure. (a) Representative images from clonogenic assays performed in shRNA control and shRNA RIG‐I transfected endothelial cells in the replicative senescence model, and the quantification of colony area and number of colonies. (b) Micrographs of cells stained for SA‐β‐Gal in cells in the Etoposide‐induced senescence model, and the quantification comparing RIG‐I deficient with control cells. (c) Growth curves for human endothelial cells cultured in vitro until they reached their Hayflick limit. (d) Quantification of secreted IL8 and IL6 in conditioned media by ELISA in the Etoposide‐induced senescence model. *N* ≥ 3, *p < 0.05; **p < 0.01, ***p < 0.001.

To test the scTenifoldKnK prediction that a RIG‐I depletion modulates senescence, we knocked down its gene *DDX58* in human endothelial cells ECFCs using an shRNA lentiviral approach. RT‐qPCR confirmed an effective *DDX58* knockdown by ~72% of basal expression level (Figure SF19a), and protein analysis corroborated the decreased RIG‐I expression (Figure SF19b). Endothelial cells with a knockdown of RIG‐I exhibited significantly higher clonogenic capacity when compared to shRNA‐controls (Figure 6a). We also found that RIG‐I deficient cells showed significantly decreased SA‐β‐Gal positivity when compared to scrambled control‐shRNA (Figure 6b). In addition, we evaluated the growth potential of endothelial cells transduced with RIG‐I‐shRNA in comparison to control‐shRNA. RIG‐I‐deficient cells exhibited significant faster growth from Day 17 onwards, which led to an extended growth curve with higher cumulative population doublings, slower doubling time lengthening, and delayed senescence establishment (Figure 6c). Importantly, knocking down RIG‐I did not lead to endothelial cell immortalization, and RIG‐I deficient cells still underwent senescence albeit with an extended in vitro lifespan. Interestingly for the same chronological age (culture passage) during the log phase of growth, RIG‐I‐deficient cells were smaller than controls (Figure SF19c). Furthermore, RIG‐I‐deficient cells exhibited reduced SASP, as demonstrated by significantly lower secretion of IL8 and IL6 (Figure 6d). In summary, RIG‐I‐deficient endothelial cells exhibited a delayed display of senescence programme coupled with increased proliferative capacity. Our data demonstrate that targeting RIG‐I is an effective strategy to extend the in vitro lifespan of endothelial cells by inhibiting the SASP and delaying senescence establishment.

## 3 DISCUSSION

Our findings demonstrate that activation of the innate immune response via RNA sensing is a hallmark of endothelial cell senescence. In the current study, using three different models of cellular senescence, we have established a new molecular fingerprint for senescent endothelium, characterized by an IFN gene signature. The EndoSEN_up gene signature is comprised of 75 genes. Because the EndoSEN signature was established using ECFCs, it is highly sensitive to detect senescent ECFCs; however, we used publicly available transcriptomics data to confirm that the EndoSEN signature is also effective to assess cellular senescence in other endothelial cell types such as HRMECs, HUVECs, and HAECs. Interestingly, the directionality of the EndoSEN signature when assessing senescent fibroblast models Wi‐38 and IMR‐90 was also positive; however, the NES were lower than 1.5. This indicates that while some genes from the EndoSEN signature were also enriched in senescent fibroblasts, as a whole 75 gene signature, the accuracy for detecting senescence was very high for endothelial cells and reduced for fibroblasts. Furthermore, our results enabled us to propose a mechanistic model whereby senescent endothelial cells display impairment in RNA metabolism leading to accumulation of self‐RNA, which activates RIG‐I sensing and triggers an IFN‐like response. This aberrant and sterile activation of innate immunity in senescent cells has been previously associated with SASP through persistent DNA damage (Rodier et al., 2009) and molecular sensing of cytosolic chromatin fragments (Dou et al., 2017). Therefore, cGAS sensing of cytoplasmic DNA has been described as essential for cellular senescence (Yang et al., 2017). Moreover, inhibition of the cGAS/STING pathway partly reversed senescence in an in vitro HAECs model (Yu et al., 2022). Similarly, STING/TBK‐1 has been shown to mediate senescence in retinal endothelial cells in a mouse diabetic model (Liu et al., 2023). Interestingly, our study did not show cGAS activation in senescent endothelial cells but found significant upregulation of RNA sensor RIG‐I and self‐RNA accumulation. This suggests that nucleic acid sensing pathways play a role in cell senescence, and while previous evidence has linked DNA sensing with senescence in endothelial cells, our results show that RNA sensing also contributes to the innate immune response occurring in senescent endothelial cells. It is likely that the involvement of DNA or RNA sensing depends on the type of senescence inducer. Our model based on mouse chronological aging did not show cGAS/STING upregulation as previously reported in diabetic retinas (Liu et al., 2023).

While there are complex checkpoints to prevent self‐recognition by RIG‐I of endogenous RNAs, accumulating evidence suggests that RIG‐I can recognize self‐RNAs and trigger sterile inflammation (Chiang et al., 2018). In addition, increased retrotransposon activity leading to LINE‐1 RNA expression in senescent cells was also shown to promote IFN activation and age‐associated inflammation (De Cecco et al., 2019). We observed enhanced RNA biogenesis and nucleoli expansion, which aligns with the theory that small nucleoli are a cellular marker of longevity (Tiku et al., 2016), and the association of nucleolar expansion with premature aging (Buchwalter & Hetzer, 2017). Similarly, cytoplasmic RNA accumulation induced by reduced RNA turnover has been described in cellular senescence (Mullani et al., 2021). This underscores the biological relevance of endogenous RNA accumulation, sensing, and consequent innate immune response in senescent endothelial cells.

We showed evidence that unequivocally links increased RNA content with endothelial senescence; however, the molecular mechanisms driving this process remain to be elucidated. Senescent cells undergo major chromatin reorganization leading to loss of heterochromatin, retrotransposon activation, and increased transcription (Criscione et al., 2016). This aligns with our finding that senescent endothelial cells exhibit increased RNA biogenesis. Another possibility is that RNA accumulates in senescent cells as the RNA degradation pathways become defective. The evident increase in cell size associated with the senescent phenotype may also explain the increased RNA content. As the cell becomes larger, its intracellular RNA content must increase to enable cell homeostasis and functionality maintenance. The current study identifies an increase in ribosomal RNA (rRNA) species in senescent cells. rRNAs constitute up to 90% of total cellular RNAs, and a study showed that dysfunction of rRNA transcription and rRNA processing induce p53 activation and senescence (Nishimura et al., 2015). Similarly, the database CSGene identified RNA polymerase I and rRNA transcription as enriched biological pathways in cellular senescence (Zhao et al., 2016). Furthermore, in a model of oncogene‐induced senescence, accumulation of rRNA precursors was found, but interestingly, this was associated with p53‐independent Rb‐dependent deficient ribosome biogenesis (Lessard et al., 2018).

Our data demonstrate that RIG‐I is an important molecular driver of endothelial cell senescence, as the RIG‐I deficient cells accumulated higher number of population doublings before reaching their Hayflick limit. This finding agrees with reports linking RIG‐I with endothelial cell dysfunction (Baris et al., 2023). Knocking down RIG‐I delayed but did not stop the cellular senescence program. Additionally, RIG‐I knockdown did not immortalize endothelial cells. These findings suggest that while RIG‐I facilitates senescence establishment, RIG‐I inhibition alone is not sufficient to prevent replicative senescence. While the role for RIG‐I in sensing viral RNA is well established (Stok et al., 2020), our findings revealed that RIG‐I also plays a role in self‐RNA sensing during endothelial senescence, and this was not observed in endothelial quiescence. Our analysis also identified IRF7 as an important transcription factor downstream of RIG‐I in senescent endothelial cells, and interestingly, epigenetic silencing of IRF7 in fibroblasts, has been suggested to be involved in the immortalization process of cells from Li–Fraumeni syndrome patients (Li et al., 2008).

Suppression of SASP constitutes a senomorphic strategy to target the detrimental paracrine effects of senescent cells. Our data showed that knockdown of RIG‐I effectively diminished SASP components IL6 and IL8. Similar results were reported in fibroblasts by targeting mTOR using rapamycin (Laberge et al., 2015). In human endothelial cells, we have previously demonstrated that IL8 deficiency extended cellular lifespan (Medina et al., 2013). Of note, it has also been shown that JAK inhibition suppressed SASP in human umbilical vein endothelial cells, and importantly, administration of JAK inhibitors to aged mice alleviated systemic inflammation and enhanced physical function (Xu et al., 2015). Furthermore, aged mouse treated with senolytic ABT‐263 showed reduced aortic stiffness and improved endothelial function mediated by SASP modulation (Clayton et al., 2023).

Our results provide new insights into the modulation of innate immune responses and the potential of novel approaches to delay aging in blood vessels. Interestingly, dietary restriction has been shown to ameliorate aging in mice by diminishing inflammation and IFN signaling activation via the Tlr/Ddx58/Ifih1‐Irf/Ifn‐Stat1 axis (Rasa et al., 2022). Understanding the molecular machinery involved in RNA biogenesis, processing, and degradation in endothelial cells, ought to facilitate the smart design of next‐generation drugs for cardiovascular aging. A question that remains unanswered is how RIG‐I senses self‐RNAs in senescent endothelial cells. It is uncertain whether this sensing is caused by RNA content overload or if senescence‐related changes to RNA biochemical structures alter the binding affinity to RIG‐I. One piece of linked evidence is that activation of the endoribonuclease RNase L cleaves self‐RNA, inducing RIG‐I, and an innate immune response (Malathi et al., 2007). In agreement with this, senescence was delayed in RNase L deficient fibroblasts (Andersen et al., 2007). Alternatively, metabolite capping of RNAs has been found to enhance RIG‐I binding affinity (Schweibenz et al., 2023). Results presented in the current study demonstrate the potential of modulating RIG‐I to appreciably delay senescence in endothelial cells using human cell models in vitro. These in vitro models are very informative for recapitulating the molecular senescence program (Chan et al., 2022), however they have limitations, and further research based on a prospective vascular aging study in living mammals would have added value. In summary, our study has established a new senescent gene signature for endothelial cells, which features a RIG‐I‐driven IFN‐like immune response. This also suggests that RIG‐I could serve as potential molecular target that could be harnessed to delay vascular aging.

## 4 MATERIALS AND METHODS

### 4.1 Cell isolation and culture

ECFCs were isolated from umbilical cord blood of full‐term pregnancies as previously described (Guduric‐Fuchs et al., 2021), with written consent from mothers and ethical approval REC 15/YH/0281. The mononuclear cell fraction was obtained by density gradient centrifugation using Histopaque (Merck) and cells were plated on collagen‐coated plates in EGM‐2 medium (Lonza) supplemented with 20% foetal bovine serum (FBS) (Gibco). MNCs were cultured for up to 4 weeks with media changes every 24 h for the first week and 48 h thereafter. ECFC colonies appeared after 7–21 days of culture. After initial colony isolation, cells were maintained in EGM‐2 supplemented with 10% FBS, and ECFC identity was confirmed by flow cytometry. Cells were passaged every 3–5 days and counted using CASY counter (Cambridge Bioscience). For replicative senescence, cells were passaged until they ceased to proliferate. We consider EP cells within 10 passages from primary isolation. Mid passage was considered between passage 15 and 20. Late passage refers to replicative senescent cells, usually between passage 25 and 30. For quiescence, cells were cultured for 5 days to reach high confluence without addition of fresh media to facilitate growth arrest. For Etoposide‐induced senescence, cells were treated with 1 μM Etoposide for 4 days, etoposide was removed, and cells were split 1:2 and kept for 4 more days for senescence establishment. For X‐ray induced senescence, cells were irradiated with 10 Gy X‐ray and kept for 4 days for senescence establishment. We defined senescent cells in this study as cells that were permanently growth arrested and expressing positivity for SA‐β‐Gal in >75% of cell population. Senescence‐associated (SA)‐β Galactosidase (Gal) staining was performed using SA‐β‐Gal Staining Kit (Cell Signaling Technology). For interferon treatment, cells were treated with 100 IU/mL Interferon‐β (R&D Systems) for 24 h. HRMECs were purchased from Innoprot and cultured on fibronectin‐coated flasks in ECM medium (Innoprot) supplemented with 5% FBS, Endothelial Cell Growth Supplements (ECGS) and penicillin/streptomycin. For replicative senescence HRMECs were passaged until reaching their Hayflick limit. We consider EP cells within the first 8 passages. Etoposide was used to induce senescence as described above.

### 4.2 Clonogenic assay

ECFCs were seeded in a 6‐well plate at a density of 200 cells per well and cultured for 10 days. ECFC‐colonies were washed with PBS before fixation and staining at room temperature for 1 h with distilled H_2_O containing 6% glutaraldehyde (vol/vol) and 0.5% crystal violet (wt/vol). Subsequently, the plate was washed with water and left to dry at room temperature.

### 4.3 Tube formation assay

Matrigel droplets were composed of growth factor‐reduced phenol free Matrigel (Corning, 356231) and ECFC cell suspension at a 1:1 ratio, respectively. 50 μL droplets were placed onto a 24‐well plate. After polymerization, EGM‐2 medium (Lonza) was added to cover Matrigel droplets. Tube‐like structures were assessed after 48 h and stained with calcein (ThermoFisher Scientific, C3100MP) for visualisation. Images were taken using a Nikon laser confocal microscope. ImageJ was used for analysis of the tube area.

### 4.4 Transcriptomic analysis

RNA for microarray was extracted from ECFCs using miRNeasy mini kit (Qiagen) and all samples had a RIN ≥8.5. Three biological replicates were used across all conditions. Microarray hybridization was performed by Arraystar using the Agilent Array platform. Further data analysis was performed using R Statistical Software (version 3.6.3; R Foundation for Statistical Computing, Vienna, Austria). Differential gene expression analysis was based on an absolute log 2‐fold change >1.0 and *p* < 0.05. RNA from HRMECs was extracted using the Maxwell® RSC instrument and simplyRNA Cells Kit (Promega). Total RNA samples were sent to Edinburgh Genomics (The University of Edinburgh) for TruSeq stranded mRNA‐seq using a NovaSeq instrument (Illumina).

### 4.5 Single cell RNA sequencing of the mouse retinal cells

Young (3 months), middle‐aged (12 months) and old (23 months) C57BL/6JRj mice were purchased from Janvier Labs, UK. All animal experiments were conducted in accordance with the UK Home Office regulations and the Association for Research in Vision and Ophthalmology. Two animals per age group were used. Two retinas were processed in one tube using the volumes as below. Therefore, four retinas from two animals per age group were pooled for sequencing. Mice were culled by CO_2_ overdose, eyes harvested, and retinas dissected. Retinas were placed in PBS supplemented with 2% FBS (vol/vol), followed by gently cutting them up using surgical scissors, and retinal tissue was pelleted at 300 g for 5 min at 4°C. Supernatant was discarded and retinal tissue was digested with 1 mL of Neurosphere Dissociation Kit (130‐095‐943, Miltenyi Biotec) dissociation solution, prepared as per manufacturer guidelines, for 5 min at 37°C on a MACSmix™ Tube Rotator (130‐090‐753, Miltenyi Biotec). This step was performed to dissociate neuronal cells and keep vascular structures intact. After digestion, tissues were pelleted at low speed of 30 rcf for 5 min at 4°C. Neuronal cells remained in the supernatant, whereas heavier vessels were pelleted. Supernatant was discarded and pelleted vessels were further digested for 15 min at 37°C using a MACSmix™ Tube Rotator. Before digestion, a small aliquot of resuspended pellet was checked under the microscope to assess for the presence of vascular structures. After pelleting at 300 g for 5 min at 4°C, digested retinal tissues were triturated in 500 μL Trituration solution prepared as previously described (Crouch & Doetsch, 2018): 50 μL of 10 mg/mL DNAse (2139, Worthington) per 1 mL of PBS plus 2% FBS (vol/vol). Tissues were triturated by pipetting up and down for approximately 30 times. After trituration, samples were spanned down at 300 g for 5 min at 4°C. Tissues were then washed two times by pelleting at 300 g for 5 min at 4°C in HGB buffer: HBSS/1% BSA (wt/vol)/0.1% D‐glucose (wt/vol). After passing cell suspension through a 40 μm cell strainer (Fisher Scientific), cells were counted. At this stage, samples obtained from retinas from each age group were pulled together for endothelial enrichment using CD31 microbeads (130‐097‐418, Miltenyi Biotec) and the autoMACS Pro Separator (Miltenyi Biotec). Endothelial cell enrichment was achieved using CD31 microbeads (130‐097‐418, Miltenyi Biotec) and the autoMACS Pro Separator (Miltenyi Biotec). Cell pellets were resuspended in 100 μL of FACS buffer (00‐4222‐26, eBioscience) containing 10 μL of CD31 MicroBeads per 10^7^ cells. After 15 min on ice cells were washed by adding 1 mL of FACS buffer and pelleting cells at 300 rcf for 10 min. Cell pellets were resuspended in 500 μL FACS buffer for magnetic separation using the autoMACS Pro Separator (Miltenyi Biotec). Cells were counted with a haemocytometer and cell suspension of 1000 cells/μL was loaded onto the microfluidic chip and libraries prepared using the 10× Genomics v3.1 kit, aiming for 5000 cell capture. Libraries were sequenced on a NextSeq 2000 (Illumina). Based on CellRanger, the number of cells obtained were 2213, 2820, and 2477 for the 3‐, 12‐, and 23‐month retinas respectively. After quality filtering, final cell counts for the analysis were 1697, 1949, and 1686, for 3‐, 12‐, and 23‐month retinas respectively. Further details and all the code used for data analysis is available at the GitHub repository https://github.com/MedinaLabUOL/AgingCellManuscript.

### 4.6 Single cell RNA sequencing of ECFCs

For single cell sequencing of cultured ECFCs, cells were used at passage 9 (proliferating), passage 19 (mid passage), and passage 26 (senescent). EP at P8 quiescent cells were grown to high confluence and cultured without media change for 5 days. Cells were detached with trypsin, washed with DPBS, passed through a 40 μm strainer and cell suspension of 1000 cells/μL was prepared and loaded onto the microfluidic chip using the 10× Genomics v2 kit, aiming for 3000 cell capture. Libraries were sequenced on NextSeq 500 (Illumina). The final cell counts after quality filtering were 2019, 1776, 726, and 1175 for the EP‐quiescent (P8), EP‐proliferating (P9), mid passage (P19) and late passage‐senescent (P26), respectively. Further details and all the code used for data analysis is available at the GitHub repository: https://github.com/MedinaLabUOL/AgingCellManuscript.

### 4.7 Interferon stimulated response element (ISRE) luciferase reporter assay

ECFCs transduced with ISRE reporter, positive, and negative control constructs (Cignal Lenti Reporter Assays, Qiagen) were treated with Etoposide for 4 days to induce senescence. Etoposide treated cells together with control untreated cells were plated in 96 well plates, 6000 cells per well and left overnight to attach. For the positive control, cells were treated with 100 IU/mL of Interferon‐β (R&D Systems) for 1 h before luciferase activity was measured using Luciferase Assay System (Promega).

### 4.8 Real‐time reverse‐transcription polymerase chain reaction

cDNA was synthesised with a High‐Capacity RNA‐to‐cDNA™ Kit (Thermo Fisher Scientific) and the real time PCR was performed using the Maxima SYBR Green qPCR mastermix (Thermo Fisher Scientific) in 10 μL reactions containing 2 μL of 1:10 cDNA dilution and 0.5 μM of gene‐specific primers for 45 cycles in a LightCycler 480 (Roche).

### 4.9 Protein extraction and Western blotting

Cells were washed once with PBS and lysed in 1× RIPA buffer supplemented with EDTA, protease and phosphatase inhibitors (Thermo Fisher). Protein extracts were quantified using the Pierce BCA Protein Assay Kit (Thermo Fisher). Twenty μg of protein was loaded onto SDS polyacrylamide gels. After electrophoresis, proteins were transferred to a PVDF membrane and blocked for 1 h in 5% Blotto Non‐Fat Dry Milk (Thermo Fisher) in TBS+ 0.1% Tween 20 (TBST), followed by incubation with primary antibodies in Clear Milk (Thermo Fisher), overnight at 4°C. The following antibodies were used: RIG‐I (MABF297, Merck), OAS1 (14498S, Cell Signaling), MDA‐5 (5321S, Cell Signaling), cGAS (15102S, Cell Signaling), STING (13647S, Cell Signaling), p21 (ab109520, Abcam), and β‐actin (5125, Cell Signaling) at 1:1000 dilution. After washing with TBST, horseradish peroxidase conjugated (HRP) secondary antibodies (Biorad) were applied at 1:3000 dilution for 1 h. Blots were developed using chemiluminescence HRP substrate (Biorad) and imaged with G:BOX instrument (Syngene). The immunoblots were quantified and analyzed using Fiji‐Image J2 software.

### 4.10 RNA selective staining via flow cytometry

ECFCs were detached and washed with PBS. Cells were stained with 500 nM SYTO™ RNASelect™ (S32703, ThermoFisher Scientific) in pre‐warmed EGM‐2 for 20 min at 37°C. Cells were washed with FACS buffer (00‐4222‐57, Thermo Fisher Scientific), centrifuged at 300 × g for 8 min at RT and resuspended in FACS buffer. 1 × 10^5^ events were recorded using the acoustic Attune NxT Flow cytometer. Unstained cells were used as control. FlowJo v10 and the CytoNorm plugin were used for analysis and batch effect correction respectively.

### 4.11 RNA extraction and quantification

For RNA measurements, total RNA was extracted using a Maxwell RSC instrument with Maxwell® RSC simplyRNA Cells Kit (Promega). To determine RNA amount per cell, RNA was extracted from 200,000 cells and quantified by Quantus Fluorometer (Promega). For RNA/DNA ratio calculations, RNA and DNA were isolated from the same cell lysate using All Prep DNA/RNA mini kit (Qiagen) and quantified with Quantus Fluorometer. RNA purity was checked using the Nanodrop (Thermo Fisher). The amount of RNA per cell was calculated by dividing the total RNA amount obtained by the number of cells used for extraction.

### 4.12 Click‐iT RNA staining

Detection of newly synthesized RNA in EP and Etoposide‐induced senescent ECFCs was performed using the Click‐iT™ RNA Alexa Fluor™ 594 Imaging Kit (Thermo Fisher Scientific). ECFCs were grown on collagen‐coated glass coverslips until confluency was reached. Cells were then stained with 1 mM 5‐ethynyl uridine (EU), prepared in EGM‐2, for 2 h in the incubator. Cells were fixed, permeabilized, and nuclei were stained following manufacturer guidelines. Cells were imaged using the EVOS cell imaging system (Thermo Fisher Scientific).

### 4.13 RNA immunoprecipitation (RIP)

RIP was performed using the Imprint® RNA Immunoprecipitation (RIP) Kit (Sigma Aldrich) following manufacturers protocol. For each RIP, 1.2 × 10^6^ of replicative senescent ECFCs were used. Immunoprecipitation was performed with anti‐RIG‐I antibody (MABF297, Merck) and anti‐SNRP70 (SAB2102255, Merck) which served as a positive control. Corresponding IgG controls were also used as negative controls. RNA from immunoprecipitated fraction was extracted using TRI reagent (Sigma Aldrich) and it was subjected to RT‐qPCR and Illumina sequencing. For the RIP qPCR, reverse transcription was performed using High‐Capacity RNA‐to‐cDNA Kit (Thermo Fisher) and PCR was carried out with Maxima SYBR green qPCR Mastermix, as described in the manual for Imprint® RNA Immunoprecipitation (RIP). Data analysis was performed using ΔΔCt method as instructed in the manual. Briefly, each RIP fraction was normalized to the input RNA fraction (1% or 100× dilution factor). ΔΔCt was calculated as ΔΔCt [RIP/NS] = ΔCt [normalized RIP] − ΔCt [normalized NS] and the Fold Enrichment = 2 (−ΔΔCt [RIP/NS]). The libraries for Illumina sequencing were constructed from total immunoprecipitated RNA fraction from each RIP, therefore starting RNA concentrations for library preparation varied and depended on the amount of precipitated RNA. The library prep was done with the KAPA RNA HyperPrep kit, without ribosomal depletion. The input was 5–10 ng per sample for RIG‐I RIP samples, and <1 ng for SNRP70 and IgG control samples. Nine PCR cycles were performed for library amplification. Samples were sequenced and the paired end reads were generated on NextSeq 2000.

### 4.14 ELISA

ECFCs were cultured for 72 h before cell culture supernatants were collected and centrifuged at 5000 g for 15 min to remove debris. Supernatants were either immediately assayed or snap‐frozen and kept at −80°C until used. ELISA kits for Interferon‐β (PBL Assay Science), IL6 and IL8 (R&D Systems) were used. Readings were obtained using the Varioskan LUX plate reader (ThermoFisher Scientific) at optical density (OD) 450 nm.

### 4.15 Immunocytochemistry

ECFCs were grown on collagen‐coated glass coverslips and fixed using 4% Paraformaldehyde for 10 min at room temperature. Blocking was performed with 5% goat serum (G9023, Sigma‐Aldrich) in 1× PBS containing 0.3% Triton X‐100 (vol/vol) for 1 h at RT. Cells were stained with primary antibody for Nucleolin (14574S, Cell Signaling, 1:1600) overnight at 4°C. Secondary antibody was applied for 1 h at room temperature. Coverslips were mounted on glass slides using Vectashield with DAPI (Vector Laboratories, H‐1200). Cells were imaged using the Leica DMi8 microscope and the Leica SP8 confocal microscope. ImageJ was used for analysis.

### 4.16 Image acquisition and analysis

SA‐β‐Gal and clonogenic images were acquired using the EVOS™ FL Auto 2 Imaging System (Invitrogen). Image analysis was performed using ImageJ and applying custom macros. Matrigel droplets were imaged using Nikon laser confocal microscopy. Analysis of tube area was carried out using ImageJ and applying custom macros. Flow cytometry data was analysed using FlowJo v10. Immunocytochemistry was imaged using the Leica DMi8 fluorescence microscope.

### 4.17 Lentiviral transduction

The lentiviral particles used were Cignal Lenti Reporter Assays (Qiagen) for ISRE reporter construct and SMARTvector lentiviral shRNA for RIG‐I (Horizon). Corresponding negative control particles were also purchased as recommended by manufacturers. ECFCs were plated in 6 well plates (50,000 cells per well) and the EGM2 media containing lentiviral particles at MOI 10 and 4 μg/mL polybrene (abm) was added to the cells. The following day, media was replaced with fresh media. Before selection, cells were expanded for 3 days, and then cultured in EGM2 containing 0.25 μg/mL puromycin for 10 days to remove cells without the construct.

### 4.18 Statistical analysis

Statistical analysis was performed using GraphPad Prism to assess differences between experimental groups. For comparisons of two groups statistical significance was determined by Student's *t* test, and the paired *t* test was used when comparing same clones across two groups. One‐way ANOVA with Bonferroni's post‐test was used for multiple comparisons. *p*‐values lower than 0.05 were considered statistically significant (ns, not significant; *p < 0.05; **p < 0.01; ***p < 0.001).

## AUTHOR CONTRIBUTIONS

J.G‐F., E.P., A.W.S., and R.J.M. designed research; J.G‐F., E.P., P.M.B., S.M.D., V.P., K.M.L., C.O.N., and R.J.M performed research; J.G‐F., E.P., P.M.B., A.W.S., and R.J.M. analyzed data; J.G‐F., E.P., A.W.S., and R.J.M. wrote the manuscript with all authors providing feedback.

## FUNDING INFORMATION

This study was supported by the BBSRC (BB/T000805/1), the Leverhulme Trust (RPG‐2015‐357), the Dunhill Medical Trust (RPGF1910\199), Diabetes UK (20/0006162), the JDRF (5‐CDA‐2014‐225‐A‐N), the Department for the Economy Northern Ireland under the US‐Ireland R&D Partnership Programme with Ref. USI 158, and the Macular Society.

## CONFLICT OF INTEREST STATEMENT

The authors declare that they have no conflicts of interest.

## Supporting information


Figure S1.

Table S1.

Table S2.

## Data Availability

The data that support the findings of this study are openly available at public repositories. Data from ECFCs treated with different senescence‐inducing stimuli were deposited on GEO (GSE160166). Data from control and senescent HRMEC cells were deposited on GEO (GSE160356). For data referring to HUVECs, HAECs, WI38 and IMR90, we used data published by Casella et al. available on GEO (GSE130727). For scRNA‐seq datasets of mouse retinas and human cells, we deposited data on GEO with GSE247586 and GSE247585, respectively. RIP‐seq data was deposited on GEO with GSE248603.
