# Supplementary material for: A new gene signature for endothelial senescence identifies self‐RNA sensing by retinoic acid‐inducible gene I as a molecular facilitator of vascular aging
Source: Aging Cell. 2024 Jun 21;23(9):e14240. doi: 10.1111/acel.14240 (PMC11488300; doi:10.1111/acel.14240)
Supplement: Supplementary file 1 — Figure S1. [file ACEL-23-e14240-s003.pdf]

Supplementary Figures

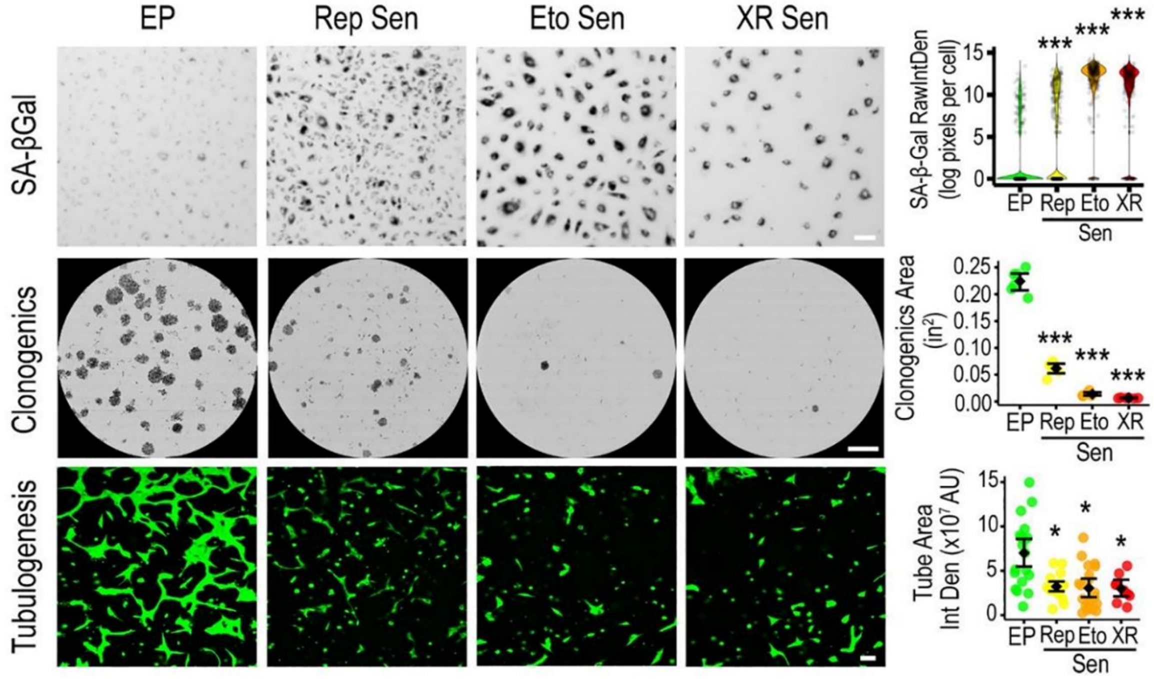

Supplementary Figure SF1: Three in vitro models of endothelial senescence. Cellular senescence was induced in early-passage (EP) ECFCs by serial passaging until cells reach their Hayflick limit (Rep Sen), or with Etoposide treatment (Eto Sen), or 10 Gy X-rays exposure (XR Sen). Senescence establishment was confirmed by SA-βGal staining (top panel), scale bar: 100 μm. Proliferative capacity was evaluated with the clonogenic assay and crystal violet staining (mid panel), scale bar: 5 mm. Endothelial function was assessed with the 3D Matrigel tube formation assay and calcein staining (bottom panel), scale bar: 100 μm. *p<0.05, ***p<0.001

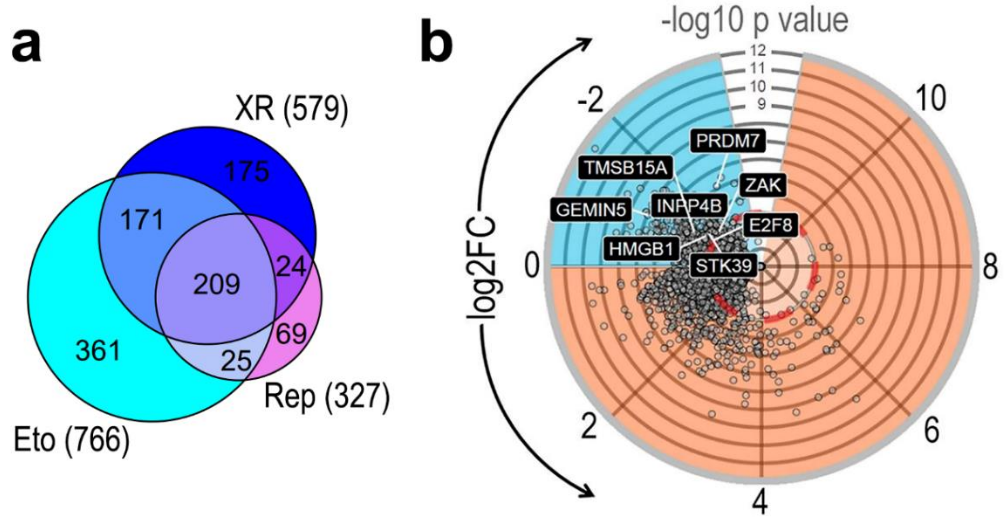

Supplementary Figure SF2: Identification of commonly downregulated genes in senescent endothelial cells. a, Euler diagram integrating lists of downregulated genes from the three cellular senescence models to characterize a common senescence signature in senescent ECFCs. **b**, Circular volcano plot depicting upregulated genes in orange and downregulated in light blue. The eight genes that are common to IFN treatment and the endothelial senescence signature have been named and highlighted in black.

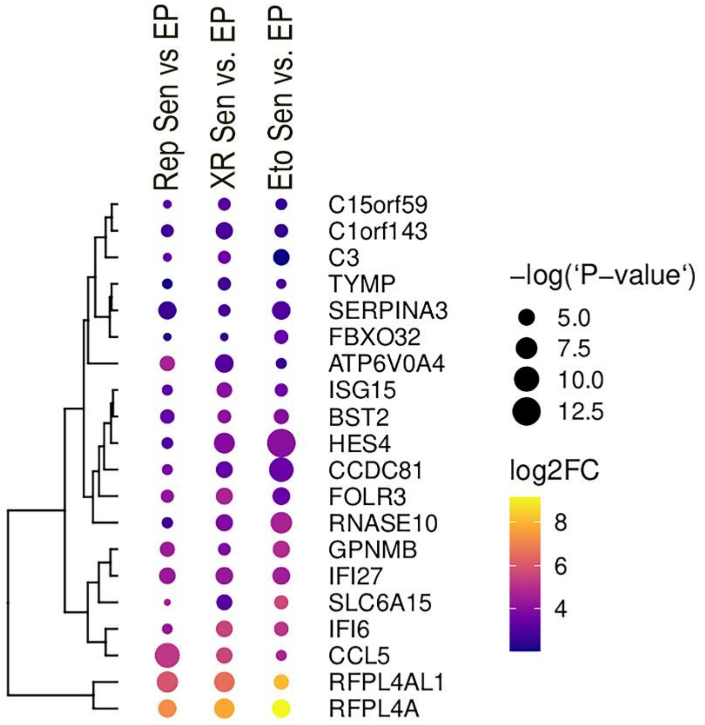

Supplementary Figure SF3: Comparison of top 20 differentially expressed genes across the 3 senescence models. Dot plot mapping the -log p value to size and log2 fold change to color for the top 20 differentially expressed genes when comparing early passage to senescent ECFCs. Each column represents the average of three biological replicates per senescence model. Rep: Replicative, XR: X rays, Eto: etoposide, EP: early passage, Sen: senescence.

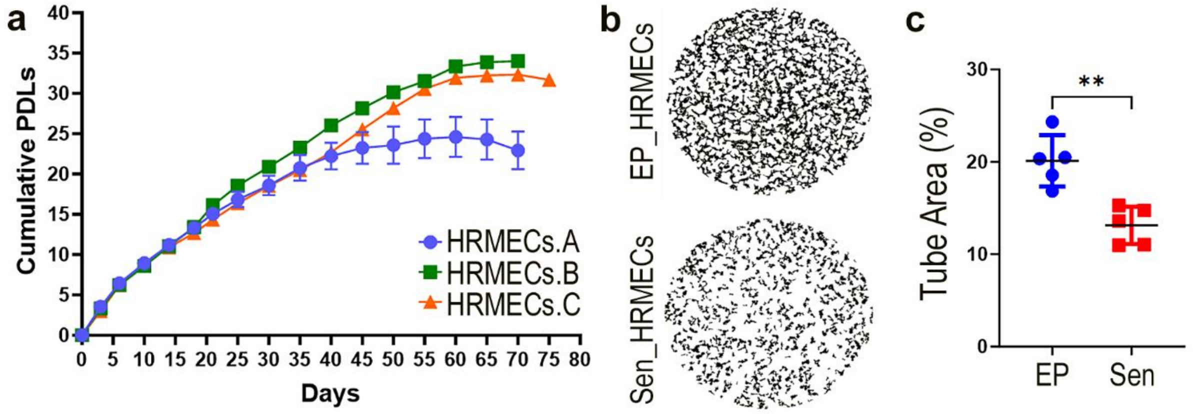

Supplementary Figure SF4: Senescence characterization in HRMECs used for the bulk RNA seq. **a**, Cellular senescence was induced in HRMECs by serial passaging until cells reach their Hayflick limit, as shown in the growth curves. Three biological replicates were used. **b**, Endothelial function was assessed with the 3D Matrigel tube formation assay and calcein staining. **c**, Quantification of HREMCs tube area as % and statistical comparison of early passage (EP) vs senescent (Sen) cells. **p<0.01.

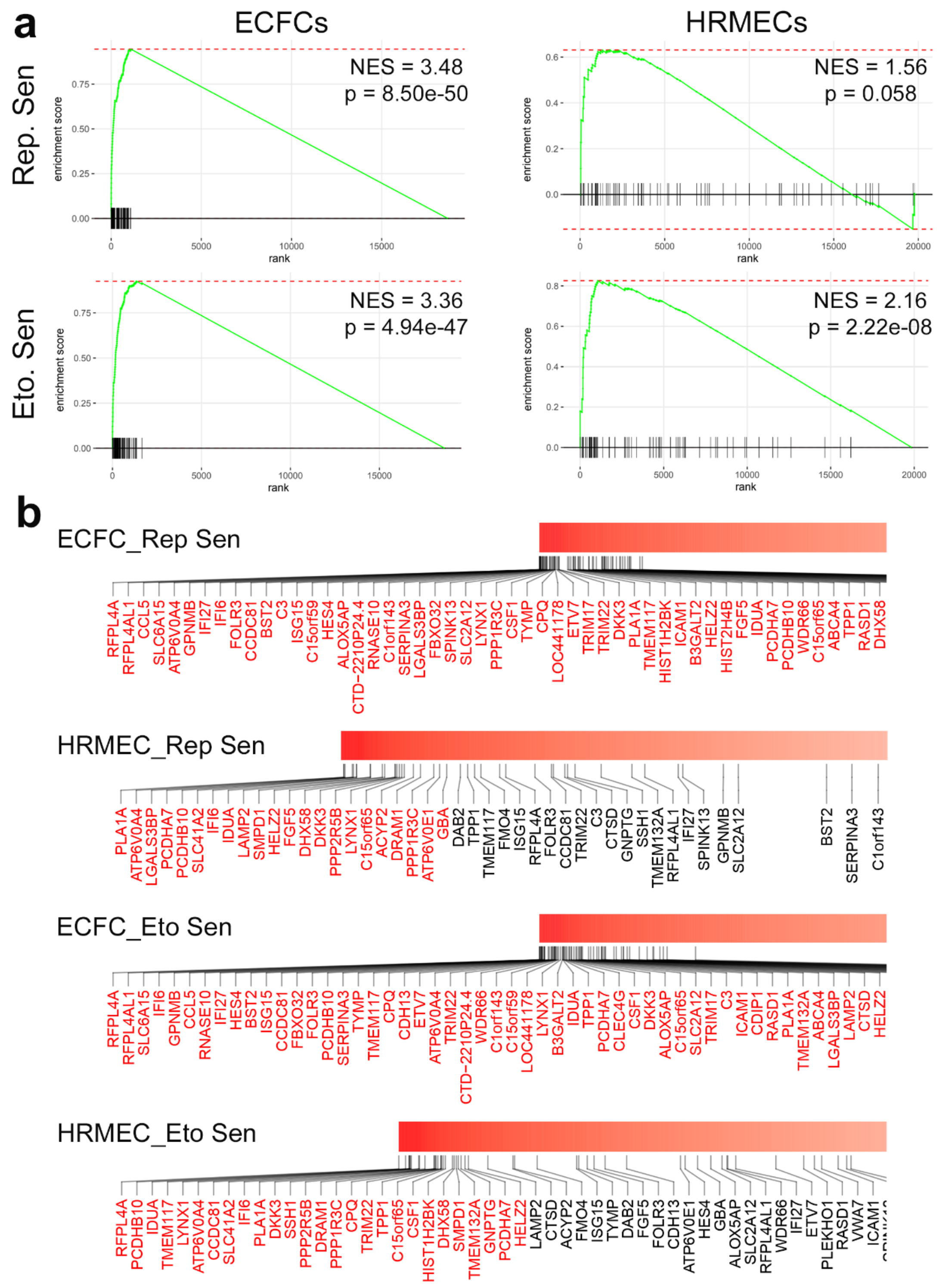

Supplementary Figure SF5: Comparison of EndoSEN_up signature in ECFCs and HRMECs. a, Gene set enrichment analysis (GSEA) plots with normalized enrichment scores (NES) and adjusted p values for the EndoSEN_up gene signature across the two senescence models in ECFCs and HRMECs. **b**, Heatmaps highlighting the ranking for the EndoSEN_up genes within the signature applied to the different experimental models. The leading edges within the GSEA, defined as the genes that contribute the most to the enrichment score, are highlighted in red.

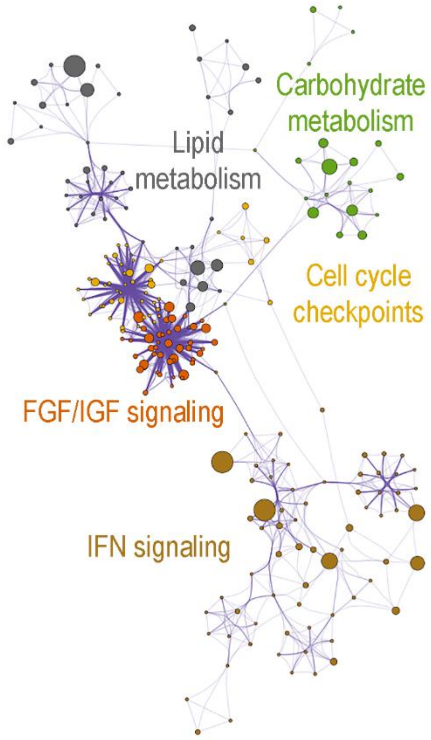

Supplementary Figure SF6: Reactome pathways highlights IFN signaling as a major cluster in senescent ECFCs. Reactome pathway network analysis from EndoSEN_up signature.

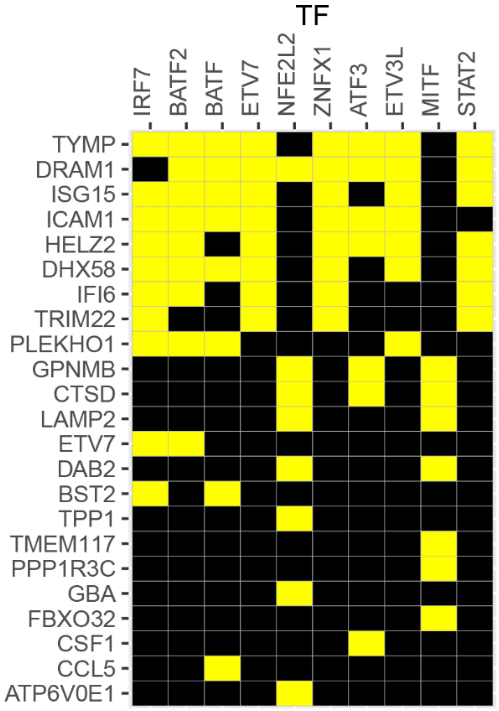

Supplementary Figure SF7: IRF7 modulates the highest number of genes from the ECFC senescent/IFN gene signature. Extended heatmap to visualise the transcription factor screening using the top 75 genes upregulated in senescent ECFCs. Yellow depicts co-expression and black no relationship.

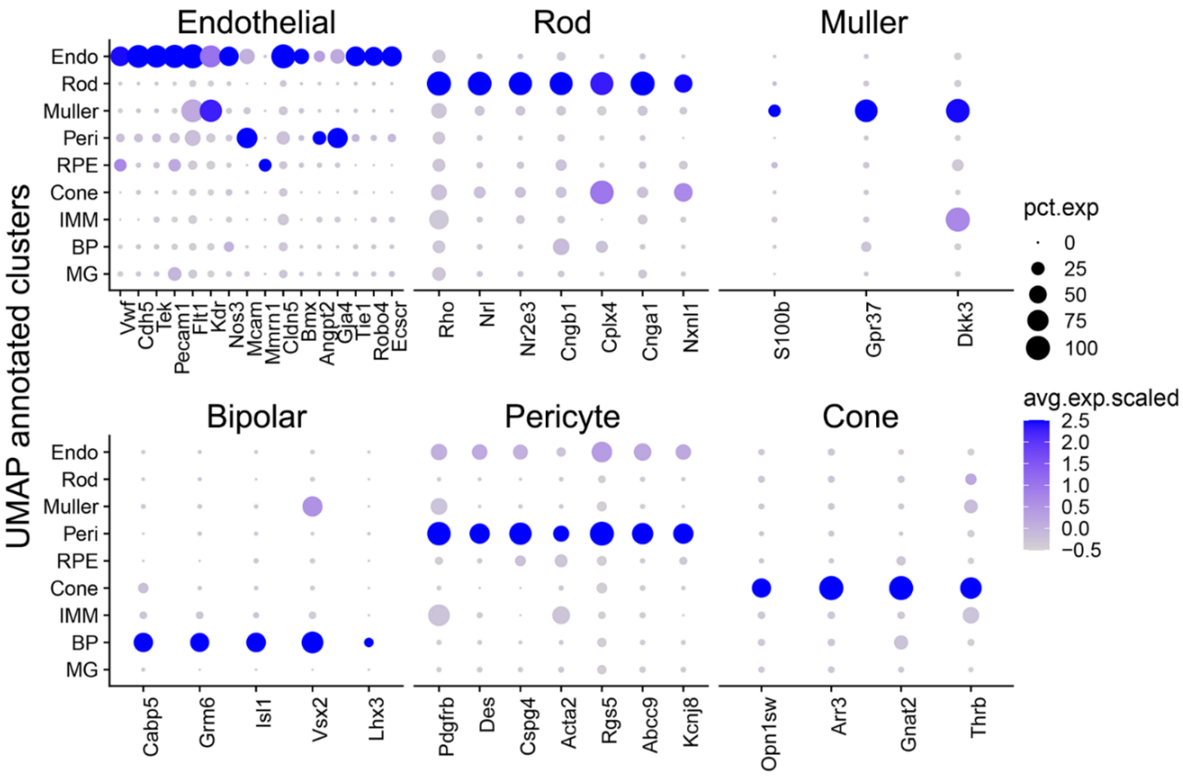

Supplementary Figure SF8: Gene signatures used for cell type annotation. Dot plot of marker genes used for cell type annotation across the 6 most frequent cell types. Annotated UMAP clusters are depicted on the Y axis. pct.exp: percentage of expression; avg.exp. scaled: average expression scaled.

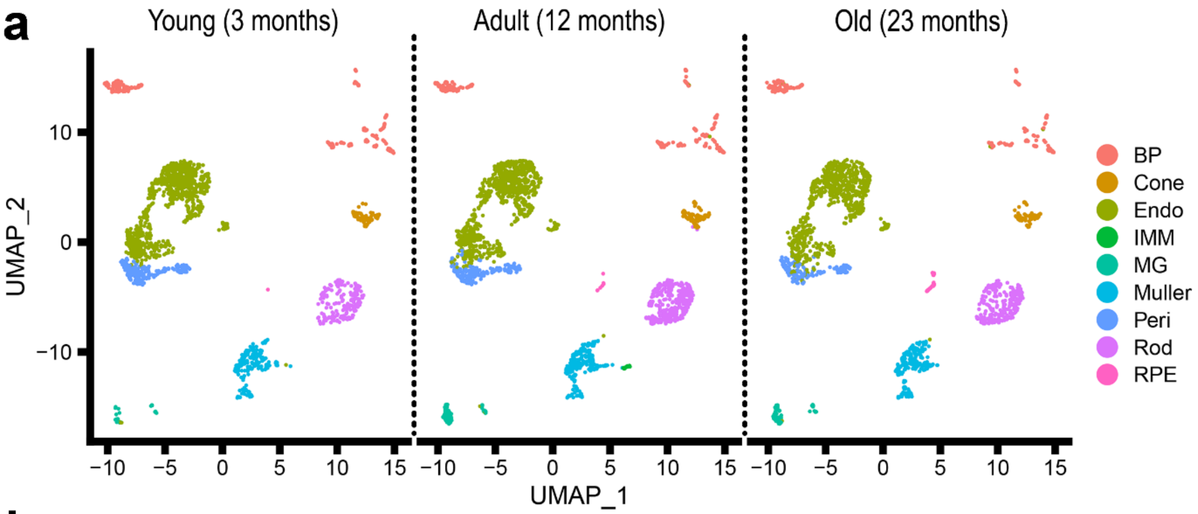

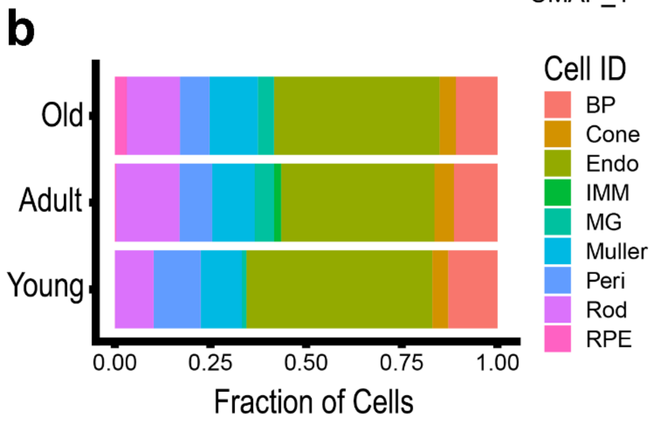

c

	Proportion (%)		
Cell ID	Young	Adult	Old
Endo	49	40	43
Rod	10	17	14
Muller	11	11	13
BP	13	11	11
Peri	12	9	8
Cone	4	5	4
MG	1	5	4
RPE	0	0	3
IMM	0	2	0
TOTAL	100	100	100

Supplementary Figure SF9: Frequency of retinal cell types exhibited minor changes with age. a, UMAP visualization of retinal cell types across the three ages studied. **b**, Barplot showing low variability of cell type proportions among the three age groups. **c**, Table depicting cell type proportions in % in young, adult, and old mouse retinas.

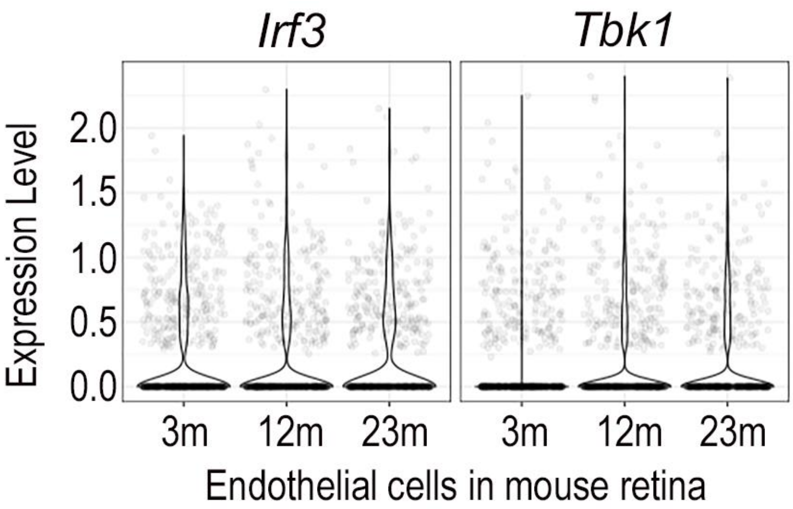

Supplementary Figure SF10: Expression level of CGAS/STING genes in mouse endothelial cells. Violin plots comparing the gene expression of *Irf3* and *Tbk1* in retinal endothelial cells among the three age groups.

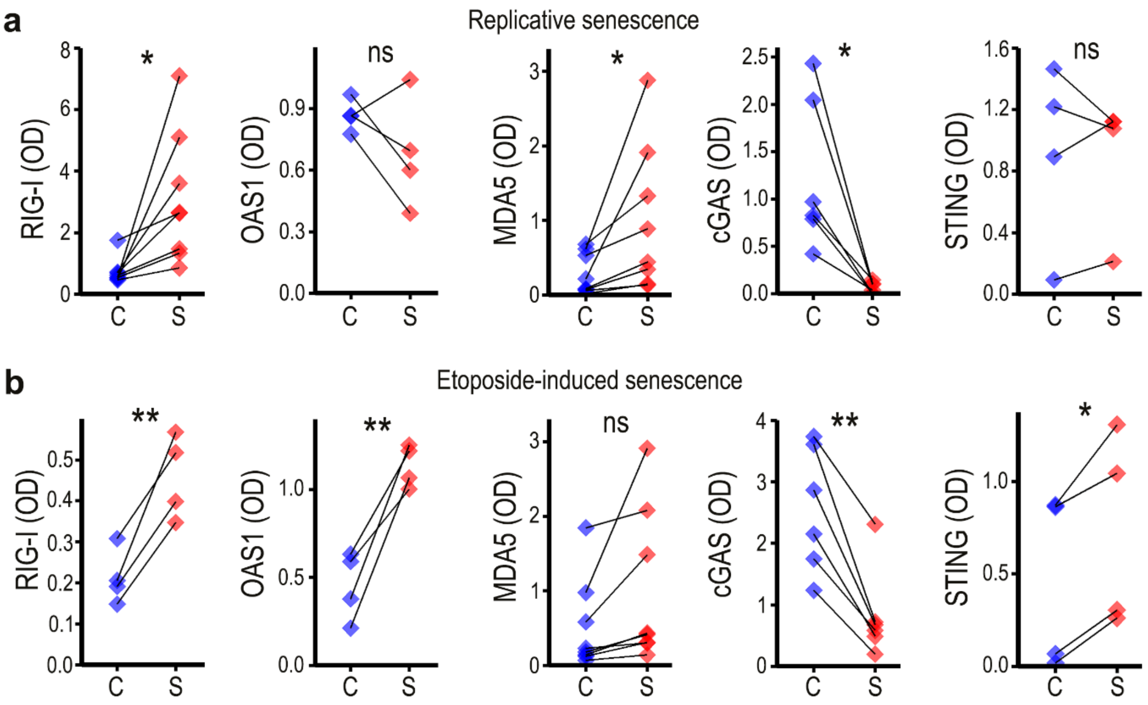

Supplementary Figure SF11: Evaluation of protein levels for RNA and DNA sensors in senescent endothelial cells. a, Quantification and statistical analysis for protein expression changes in the replicative senescence model. OD=Optical density **b**, Quantification and statistical analysis for protein expression changes in the Etoposide-induced senescence model. N=4-8 biologically independent samples, ns: not significant, *p<0.05; **p<0.01.

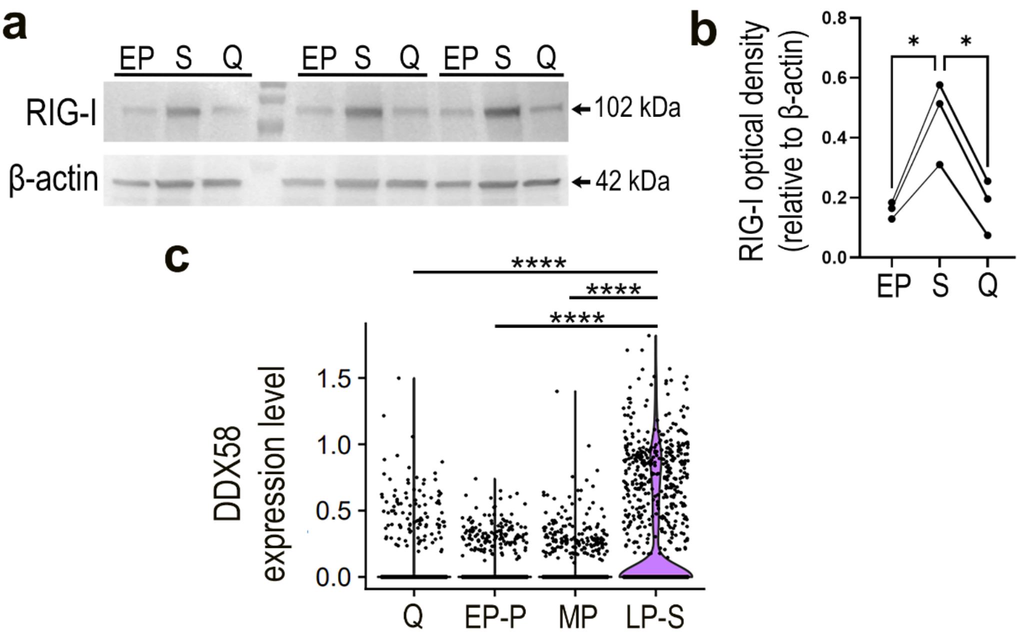

Supplementary Figure SF12: RIG-I is not upregulated in quiescent endothelial cells. a, Western blot analysis of RIG-I expression in early passage (EP), Etoposide-induced senescent (S), and quiescent (Q) ECFCs in three biological replicates. **b**, Optical density quantification and statistical analysis for RIG-I protein expression changes, *p<0.05. **c**, Violin plots to visualize *DDX58* (RIG-I coding gene) in scRNAseq dataset including quiescent (Q), early passage proliferating (EP-P), mid passage (MP), and late passage senescent (LP-S) ECFCs. ****p<0.0001.

a

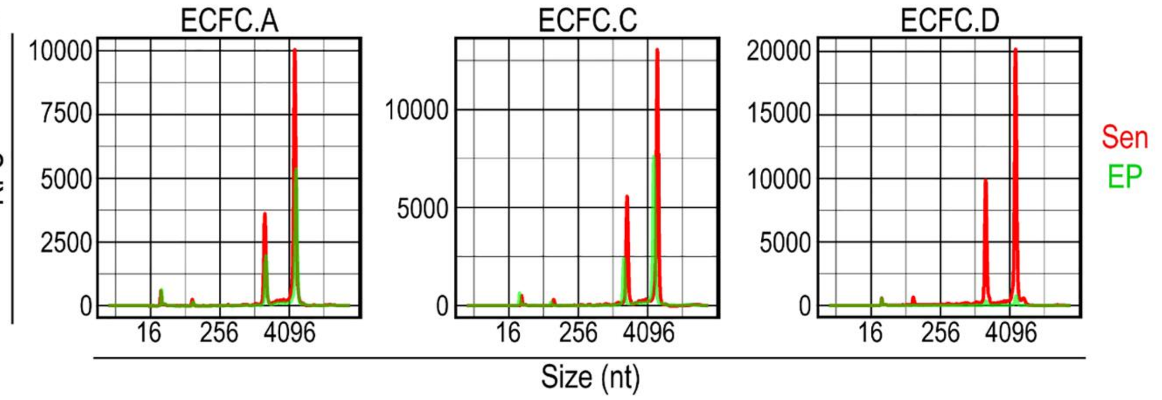

b

RIP-SEQ FEATURE COUNTS AFTER MULTIMAPPING FOR RIBOSOMAL RNAs			
**Sample ID**	**Total Reads**	**Condition**	**Size Factor**
RIP_RIGI.01	46395367.62	RIP_RIGI	2.496731847
RIP_RIGI.02	45652411.69	RIP_RIGI	2.302100209
RIP_RIGI.03a	41145959.40	RIP_RIGI	1.371731608
RIP_RIGI.03b	47884551.86	RIP_RIGI	1.656802596
RIP_SNRNP70.03	3070752.03	RIP_SNRP70	0.26429214
IgG_CONTROL.03	4548506.99	RIP_IGG CONTROL	0.327531065

c

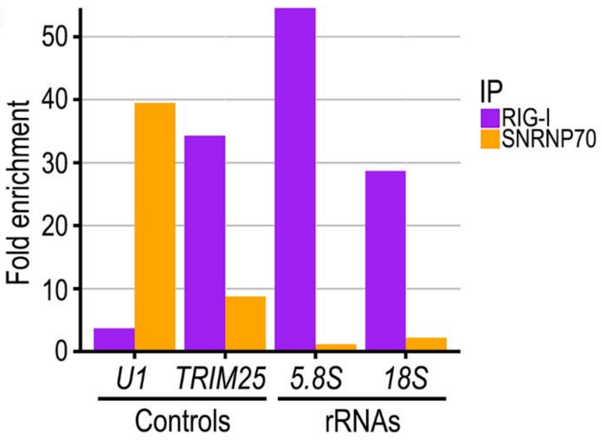

Supplementary Figure SF13: Senescent ECFCs showed higher intracellular RNA content than early passage cells. a, Extended data for Figure 4i, showing similar results in three additional biological replicates. Electropherograms for total RNA isolates from 200,000 cells comparing senescent (Sen) ECFCs in red with early passage (EP) counterparts in green. **b**, Total Read Counts from RIP-Seq raw data representing any mapped transcript. A multimapping approach was followed to include rRNAs. The original input was the same number of cells. **c**, RIG-I directly binds to rRNAs. Quantification of immunoprecipitated RNA was performed by qPCR. U1 snRNA, TRIM25 mRNA, and 5.8S and 18S rRNAs were amplified using template RNA immunoprecipitated with antibodies against RIG-I and SNRNP70. Each RIP RNA fractions were normalized to the input RNA fraction and the fold enrichment was calculated as FE = 2 exp (-ΔΔCt [RIP/NS]) where NS is nonspecific background immunoprecipitated with IgG control antibody.

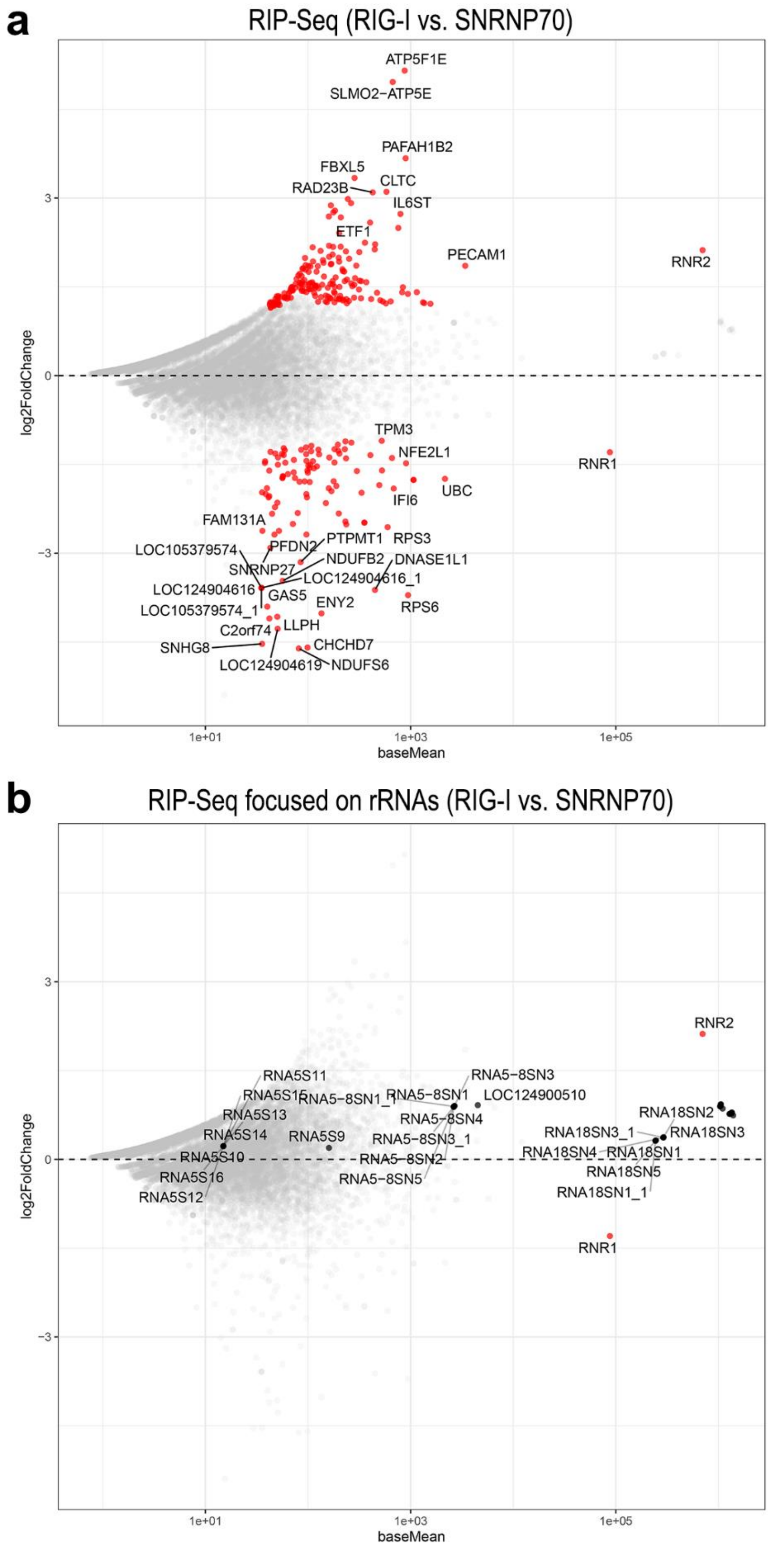

Supplementary Figure SF14: Differential RNA binding characterized by RIP-Seq. Statistically significant differences are highlighted in red. **a**, MA plot to depict log2 fold-change versus mean expression from RIP-Seq to compare RNAs that bind to RIG-I and SNRNP70. **b**, MA plot focused on ribosomal RNA biotype.

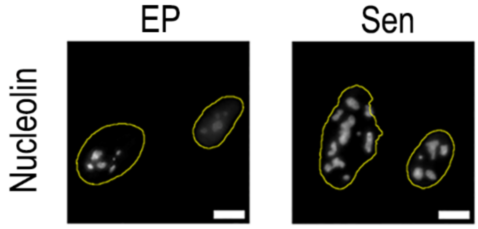

Supplementary Figure SF15: Increased Nucleolin expression in senescent endothelial cells. Representative images of Nucleolin immunostaining in early passage (EP) and senescent (Sen) endothelial cells. Scale bar: 10 µm.

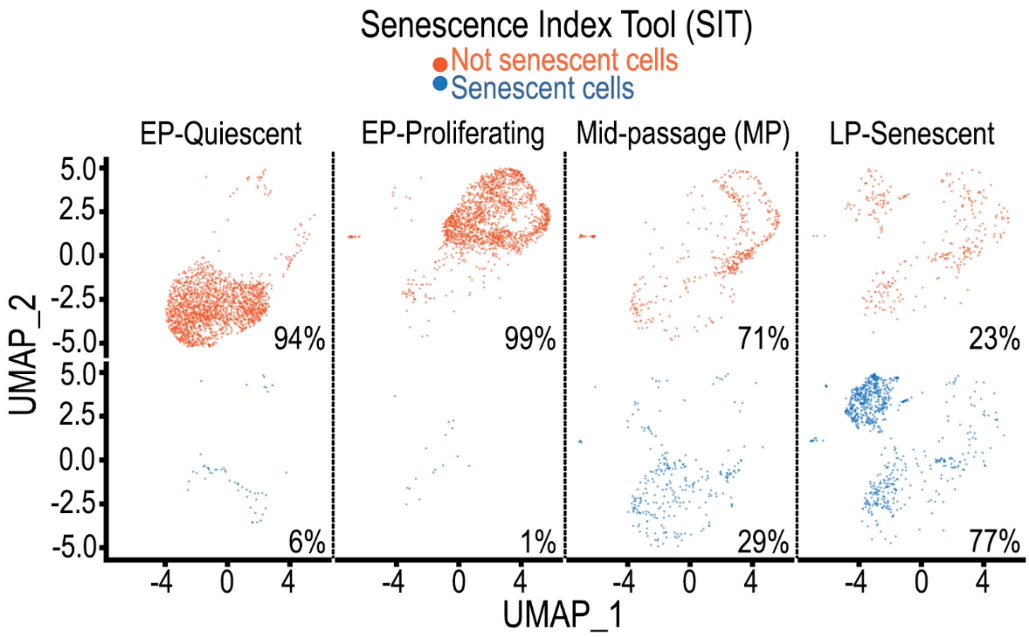

Supplementary Figure SF16: Senescence Index Tool algorithm quantifies senescent cell proportion in experimental groups and identifies cluster enriched for senescent cells.

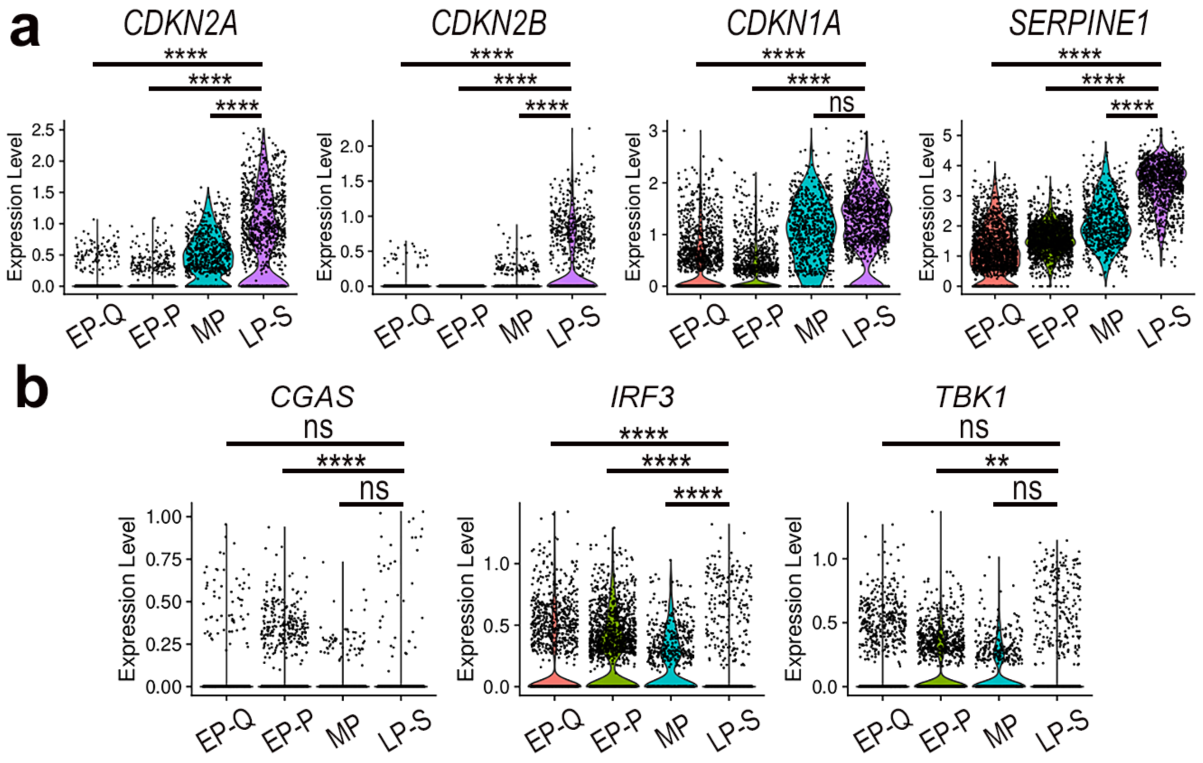

Supplementary Figure SF17: Expression levels of selected senescence and cGAS genes from scRNAseq data for the in vitro aging model in human cord blood ECFCs. a, Violin plots comparing the gene expression of prototypical senescence genes *CDKN2A* (p16), *CDKN2B* (p15), *CDKN1A* (p21), and *SERPINE1* (PAI1) across all experimental groups. **b**, Violin plots for cGAS/STING signature genes *CGAS*, *IRF3*, and *TBK1* in ECFCs. Early passage-quiescent (EP-Q), early passage-proliferating (EP-P), mid passage (MP) and late passage-senescent (LP-S). ns: not significant, **p<0.01, ****p<0.0001.

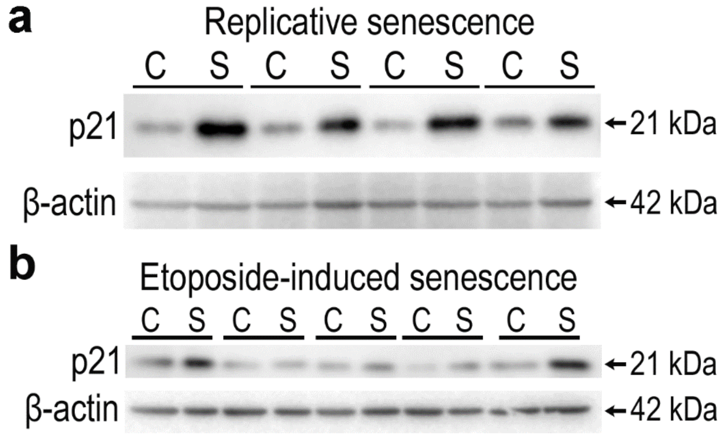

Supplementary Figure SF18: Evaluation of p21 in senescent ECFCs. a, Western blot for p21 in control non-senescent (C) and replicative senescent (S) ECFCs. **b**, Western blot assessing p21 expression in Etoposide-induced senescent ECFCs (S) compared to their non-senescent controls (C).

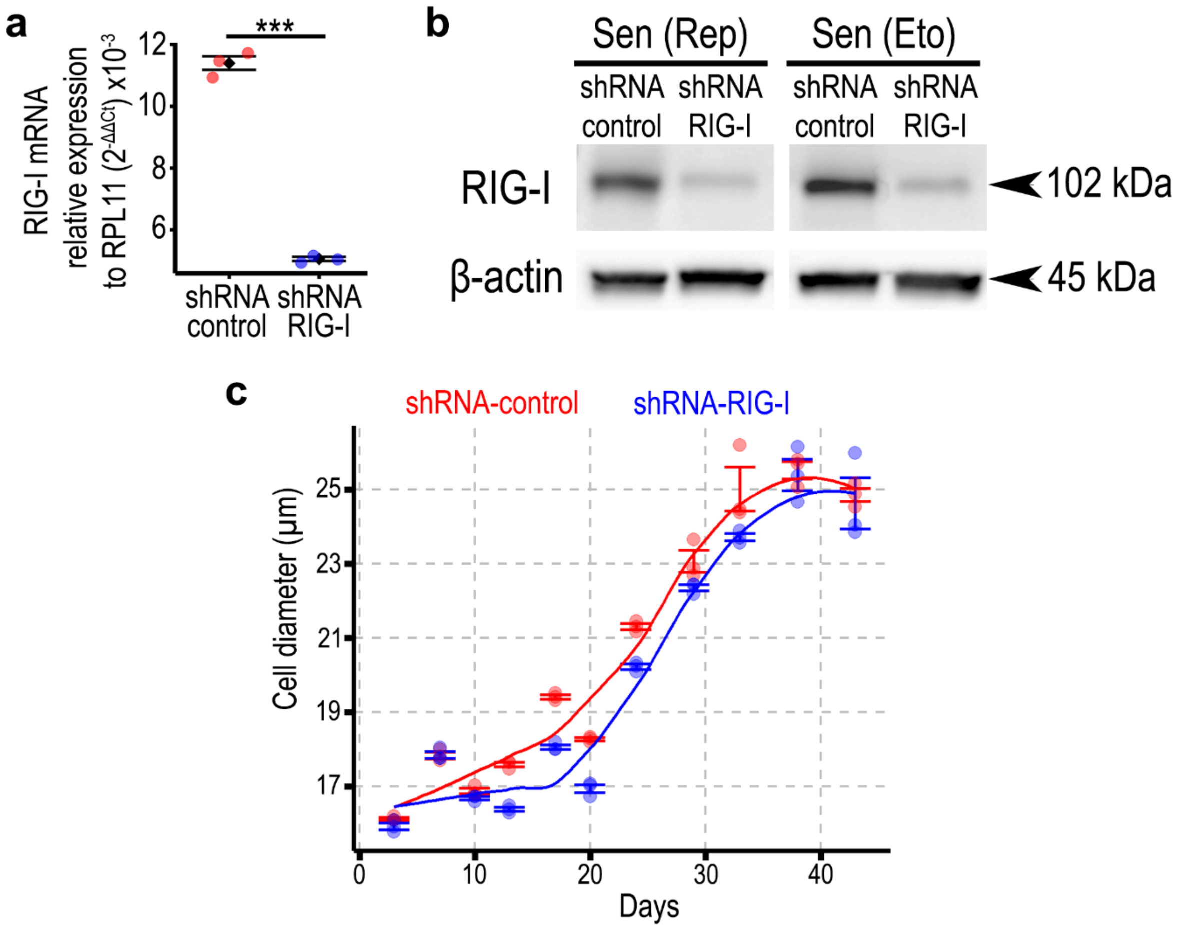

Supplementary Figure SF19: Effective RIG-I knockdown in ECFCs. a, Evaluation of RIG-I knockdown by RT-qPCR in senescent endothelial cells. *** p<0.001. **b**, Expression level of RIG-I assessed by Western blotting in shRNA-control or shRNA-RIG-I transfected cells in the replicative (Rep) and etoposide-induced (Eto) senescence models. **c**, Scatter plot to visualize cell diameter changes measured by the CASY system in µm for human endothelial cells across their in vitro lifespan. ECFCs deficient in RIG-I (shRNA-RIG-I) in blue, are compared to controls (shRNA-control) in red.
